# Comprehensive Analysis of the Expression and Prognosis for ITGBs: Identification of ITGB5 as a Biomarker of Poor Prognosis and Correlated with Immune Infiltrates in Gastric Cancer

**DOI:** 10.3389/fcell.2021.816230

**Published:** 2022-02-09

**Authors:** Dongliang Liu, Shaojun Liu, Yu Fang, Liu Liu, Kongwang Hu

**Affiliations:** ^1^ Department of General Surgery, The First Affiliated Hospital of Anhui Medical University, Hefei, China; ^2^ Department of General Surgery, The First Hospital Affiliated to the University of Science and Technology of China, Hefei, China

**Keywords:** ITGBs, gastric cancer, ITGB5, prognosis, immune infiltrate

## Abstract

**Background:** Integrin β superfamily members (ITGBs) are documented to play important roles in various biological processes, and accumulating evidence suggests that ITGBs are associated with carcinogenic effects in several malignancies. Gastric cancer (GC) is a complicated and highly heterogeneous disease; however, the expression and prognostic values of eight ITGBs and potential mechanism in GC remain largely unclear.

**Methods:** The expression and prognostic significance of ITGBs in GC were systematically analyzed through Gene Expression Profiling Interactive Analysis, Human Protein Atlas, Kaplan–Meier Plotter, and cBioPortal databases. Then, the mRNA transcription data and corresponding clinical data of GC were downloaded from the Gene Expression Omnibus database as a testing cohort, and differentially expressed and prognostic genes were identified. The correlation between ITGB5 expression and overall survival and various clinical parameters were found by using univariate/multivariable Cox regression and Kaplan–Meier survival analysis. Additionally, differential analysis of gene expression profiles in low- and high-ITGB5 expression groups and pathway enrichment analysis was performed. Finally, the correlation of ITGB5 expression with immune infiltrates in GC was clarified.

**Results:** Compared with adjacent normal tissue, the results reveal that the mRNA levels of ITGB1-2 and ITGB4-8 are significantly higher in GC, and immunohistochemistry results show the consistency between RNA and protein expression levels. Cox regression and Kaplan–Meier survival analysis indicate that high ITGB5 expression contributes to a poor prognosis and could be an independent prognostic factor in GC patients. Besides this, gene functional enrichment analysis indicates that ITGB5 expression is significantly associated with extracellular matrix organization, cell-substrate adhesion, and ossification. The KEGG pathway analysis of ITGB5 shows a close association between ITGB5 and focal adhesion, ECM-receptor interaction, phagosome, and PI3K-Akt signaling pathway. Last, the infiltrating level of CD4^+^ T cells, macrophages, and dendritic cells are positively related to the expression of ITGB5, especially macrophages, and lower levels of macrophages predict a better prognosis in GC in our study.

**Conclusion:** Our findings investigate that ITGB5 may function as a valid biomarker of prognosis, and high expression of ITGB5 predicts poor prognosis for patients with GC. Besides this, it might be a potential target of precision therapy against GC.

## Introduction

Gastric cancer (GC) is the fifth most common cancer and the third leading cause of cancer-related deaths globally (Ferlay et al., 2019). Despite the improvement in radiological diagnosis and surgical techniques, a low 5-year survival rate of patients with advanced GC remains a challenge because of the late presentation, high metastasis, and recurrence rate. The American Joint Committee on Cancer (AJCC) TNM staging system, which is based on tumor infiltration depth (pT), number of lymph node metastases (pN), and the presence of distant metastasis are the main reference index for predicting the prognosis of patients (Compton, 2007). The 8th edition of the AJCC TNM staging system for GC was published in October 2016 and officially implemented in January 1, 2018, in which the N3a and N3b categories were separately introduced into different TNM subgroups (Ilhan et al., 2016). However, due to epigenetic changes, multiple genetic alterations, and the tumor microenvironment, GC is a complicated and highly heterogeneous disease and results in variable prognosis in patients. In addition, there are still significant differences in the survival outcomes of patients with the same clinicopathological characteristics, which means that the current TNM staging system cannot reflect the intrinsic tumor heterogeneity. Hence, further exploration of a specific biomarker is an unmet medical need for improving the diagnosis and prognosis of GC.

The family of integrin is a transmembrane glycoprotein widely existing in the cell membrane, and it consists of α and β subunits by noncovalent bonds. To date, eight members of the Integrin β (ITGB) family of proteins have been identified in organisms. Integrin has a two-way signal transduction function due to its particular transmembrane structure, which interacts with the extracellular matrix (ECM) to activate related signaling pathways and play a pivotal role in the regulation of various biological behavior, including proliferation, adhesion, migration, and differentiation (Chung and Kim, 2008; Ginsberg, 2014; Bianconi et al., 2016). Deregulation of integrin signaling is reported to associate with carcinogenic effects in several malignancies (Shen et al., 2019; Song et al., 2020). Lin et al. (2018) reports that ITGB5 is highly expressed in hepatocellular carcinoma (HCC), and miR-185 regulates the expression of β-catenin through the ITGB5-dependent manner and affects the proliferation and migration of HCC cells. In pancreatic cancer, ITGB4 is demonstrated to be associated with epithelial-mesenchymal transition. Overexpression of ITGB4 promotes pancreatic carcinogenesis and regulates the MEK1-ERK1/2 signal pathway (An et al., 2016). Cui et al. (2018) found that ITGB8 promotes ovarian carcinogenesis, and overexpression of ITGB8 was associated with drug resistance. Similar findings are observed in other types of cancer (Laudato et al., 2017; Huang et al., 2019; Shen et al., 2019). However, the prognostic value and potential biological functions of the entire ITGBs in GC are still largely elusive.

Herein, this study aimed to identify the expression and prognostic values of the eight ITGBs and search for the potential therapeutic biomarker of GC patient survival. Furthermore, we explore the underlying mechanisms based on ITGB5-related GC genes using a pathway enrichment analysis. Finally, the correlation of ITGB5 expression with immune infiltrates in GC is clarified by Tumor IMmune Estimation Resource (TIMER).

## Materials and Methods

### Data Download and Preprocessing

The expression and prognostic significance of ITGBs in GC were systematically analyzed through Gene Expression Profiling Interactive Analysis (GEPIA) (http://www.gepia.cancer-pku.cn/http://www. gepia.cancer-pku.cn/) and cBioPortal databases (https://cbioportal.org). The expression of ITGBs in GC was displayed using boxplots with statistical significance evaluated using the Wilcoxon test and marked with an asterisk. Correlation between mRNA expression of ITGBs and tumor stages in patients with GC was generated and displayed, and the Kaplan–Meier curves were plotted to assess the survival and prognostic values of ITGBs. Data from cBioPortal was selected to analyze genetic changes of gastric cancer, and genetic alterations among diverse types of GC were shown in different colors.

### Immunohistochemical Staining Evaluation

The Human Protein Atlas (HPA) (https://www.proteinatlas.org/) aimed to map all the human proteins in cells, tissues, and organs using an integration of various -omics technologies, and consists of six separate parts, each focusing on a particular aspect of the genome-wide analysis of the human proteins. In this study, immunohistochemistry (IHC) images of the ITGBs protein expression in clinical samples of patients with normal and GC tissues were searched in HPA.

### Identification of Differentially Expressed and Prognostic Genes

The mRNA transcription data and corresponding clinical data of GC were downloaded from the GEO database as a testing cohort, and the data set with fewer than 60 samples or with incomplete follow-up information was excluded from our selection. Age, overall survival (OS), gender, grade, and TNM stage were obtained. Subsequently, the gene expression profile (GSE84437) was filtered from theGEO database, which contained 357 GC tissues. We combined all this information into a matrix file using Perl language (http://www.perl.org/). Transcriptome data were subjected to differential analysis, and differentially expressed and prognostic genes were identified based on the GEO database using the “survival package” and “limma package” in R software.

### Prognosis Analysis and the Association of ITGB5 Expression with Clinical Features in GC

Univariate/multivariable Cox regression and Kaplan–Meier survival analysis were used to analyze the relationships between low- and high-ITGB5 expression and OS rate and various clinical features. The prognostic value of ITGB5 mRNA expression was verified using an online database, Kaplan–Meier Plotter (www.kmplot.com), which contains survival information and expression data of GC patients.

### Functional Enrichment Analysis and Immune Cell Infiltration

Functional enrichment analysis was conducted in R studio to identify important key genes and significantly enriched pathways involved in oncogenesis and tumor progression of GC. The heatmap and volcano plot of the differentially expressed genes were drawn using the “pheatmap package” and “ggplot2 package.” Then, we transformed the gene symbols into gene IDs *via* the “Biomanager” and “org. Hs.eg.db” package. Pathway enrichment analysis based on the Gene Ontology (GO) and Kyoto Encyclopedia of Genes and Genomes (KEGG) pathway by using the “ggplot2,” “cluster Profiler,” and “enrich plot” packages. Furthermore, we explore the correlation between immune cell infiltration and ITGB5 in GC by using the “gene” and “survival” modules in TIMER.

### Statistical Analysis

R version 4.0.5 and Perl version 5.28 were used to complete the statistic work. The Kaplan–Meier method with the log-rank test was used to analyze the survival rate. The univariate/multivariate Cox proportional hazard regression model was used to analyze the significant transcription factors affecting OS. *p* values <.05 indicate statistical significance.

## Results

### Transcriptional Levels of ITGBs in Patients with GC

**FIGURE 1:**
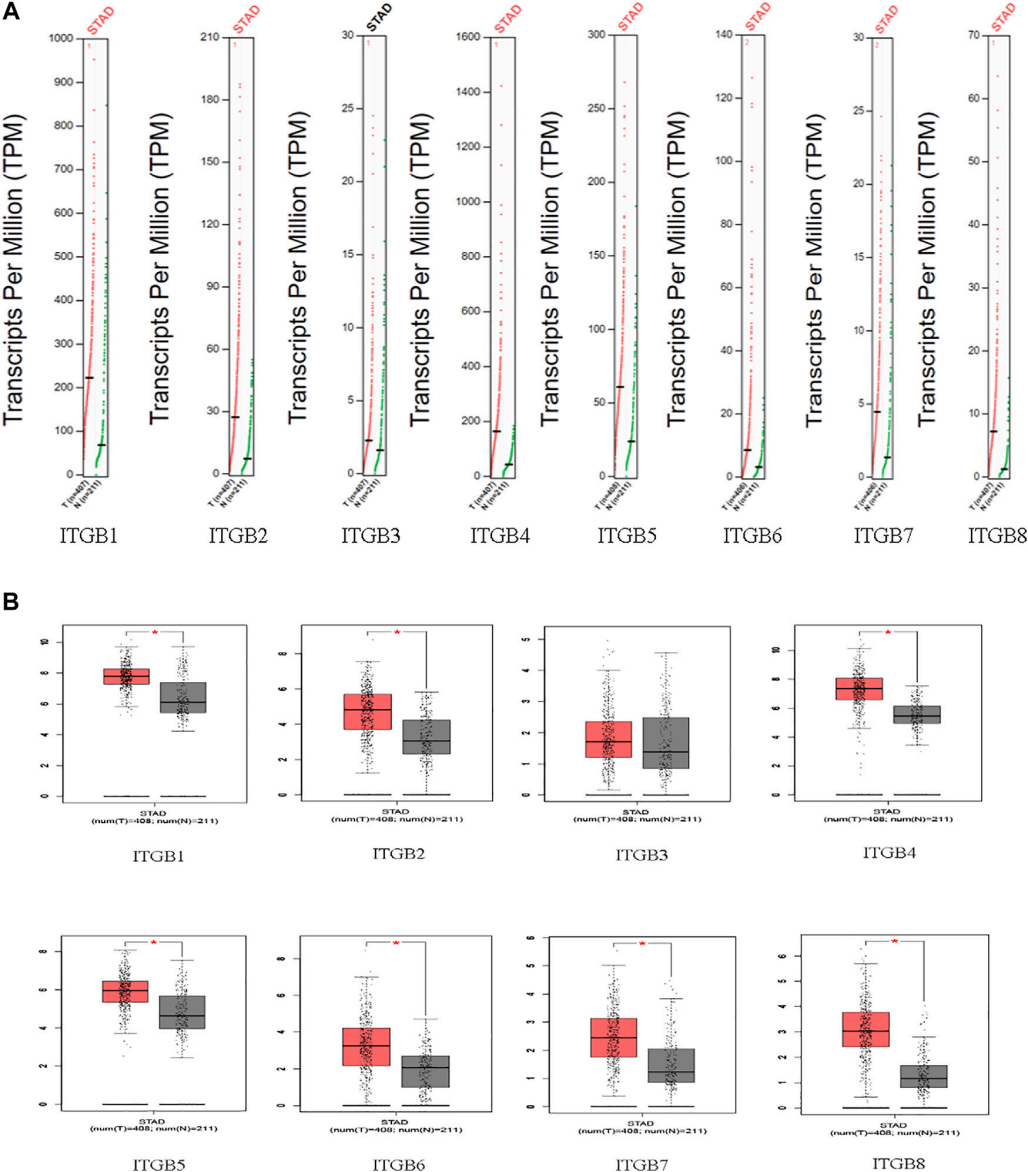
The expression of ITGBs in GC [**(A)** scatter diagram; **(B)** box plot] (GEPIA).

The mRNA expression of ITGBs in normal and GC tissues was analyzed using GEPIA. Based on a wide variety of data sets, the results reveal that the mRNA levels of ITGB1, ITGB2, ITGB4, ITGB5, ITGB6, ITGB7, and ITGB8 were significantly higher in GC than in normal tissues. Besides this, ITGB3 was confirmed with a similar expression in GC compared with normal tissues (Figures 1A,B).

### Correlation Between mRNA Expression of ITGBs and Pathological Stages in Patients with GC

**FIGURE 2:**
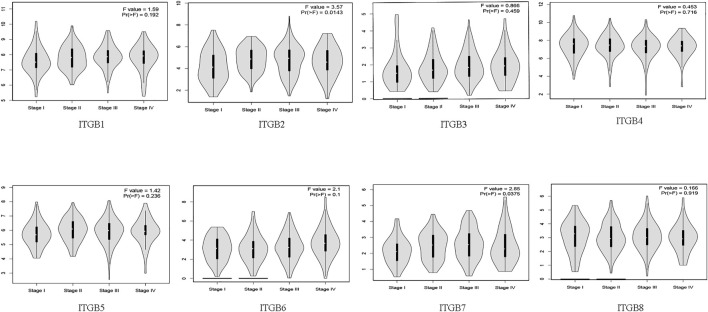
Correlation between mRNA expression of ITGBs and tumor stages in patients with GC (GEPIA).

The results indicate that significant statistical differences between tumor stages I–IV were identified in the ITGB2 and ITGB7 groups. There was no association between the other ITGB members and pathological stage (*p* > .05; Figure 2).

### Survival Analysis and Prognostic Values of ITGBs in Patients with GC

**FIGURE 3:**
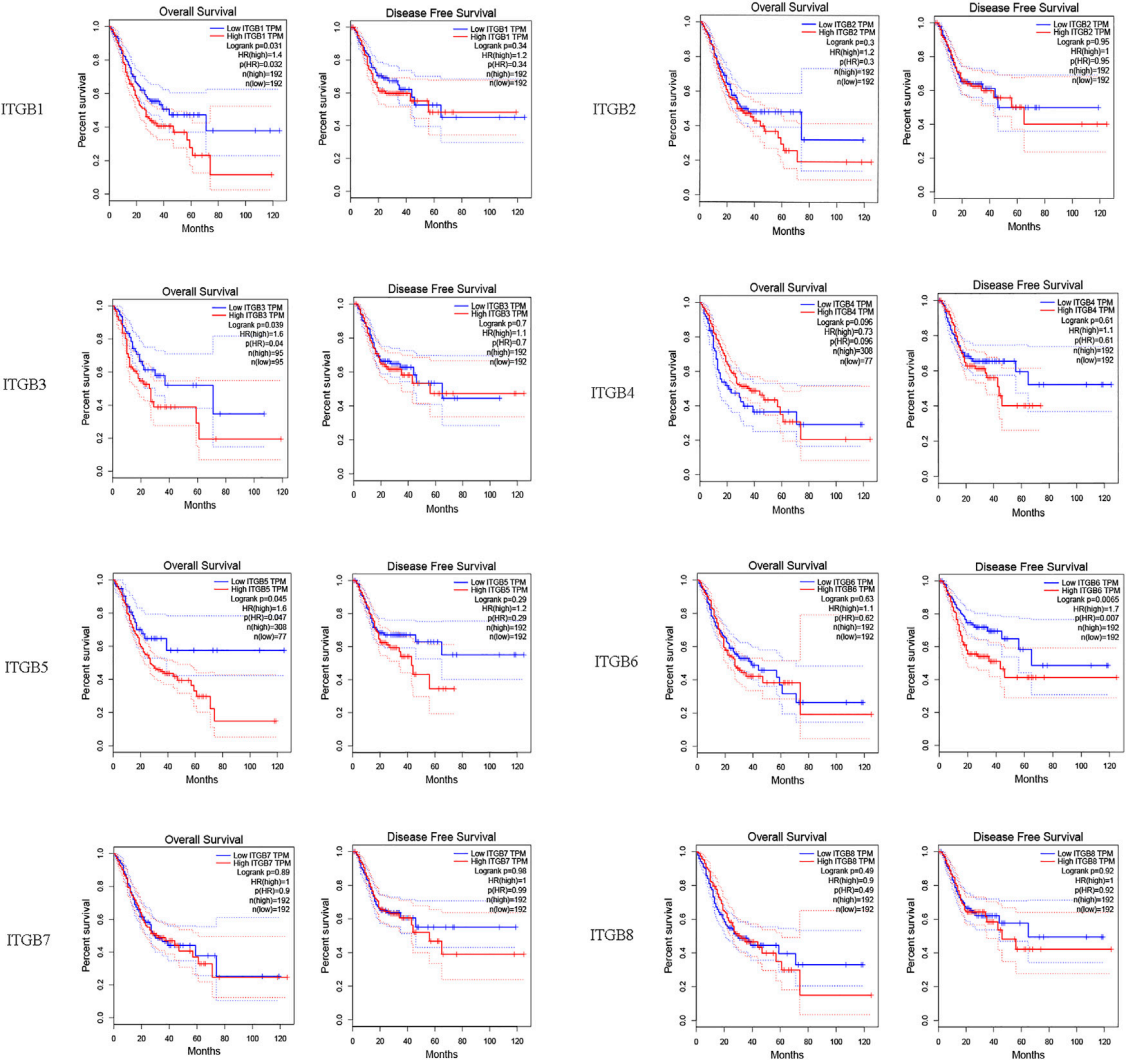
Survival analysis and prognostic values of ITGBs in patients with GC (GEPIA).

We investigated correlations between ITGB expression levels and patient prognosis using GEPIA. The results reveal that the expression levels of ITGA1, ITGA3, and ITGB5 were remarkably correlated with OS in GC patients (Figure 3). However, no significant difference was observed in DFS in most ITGB family members except ITGB6.

### IHC Analysis of ITGBs in GC

**FIGURE 4:**
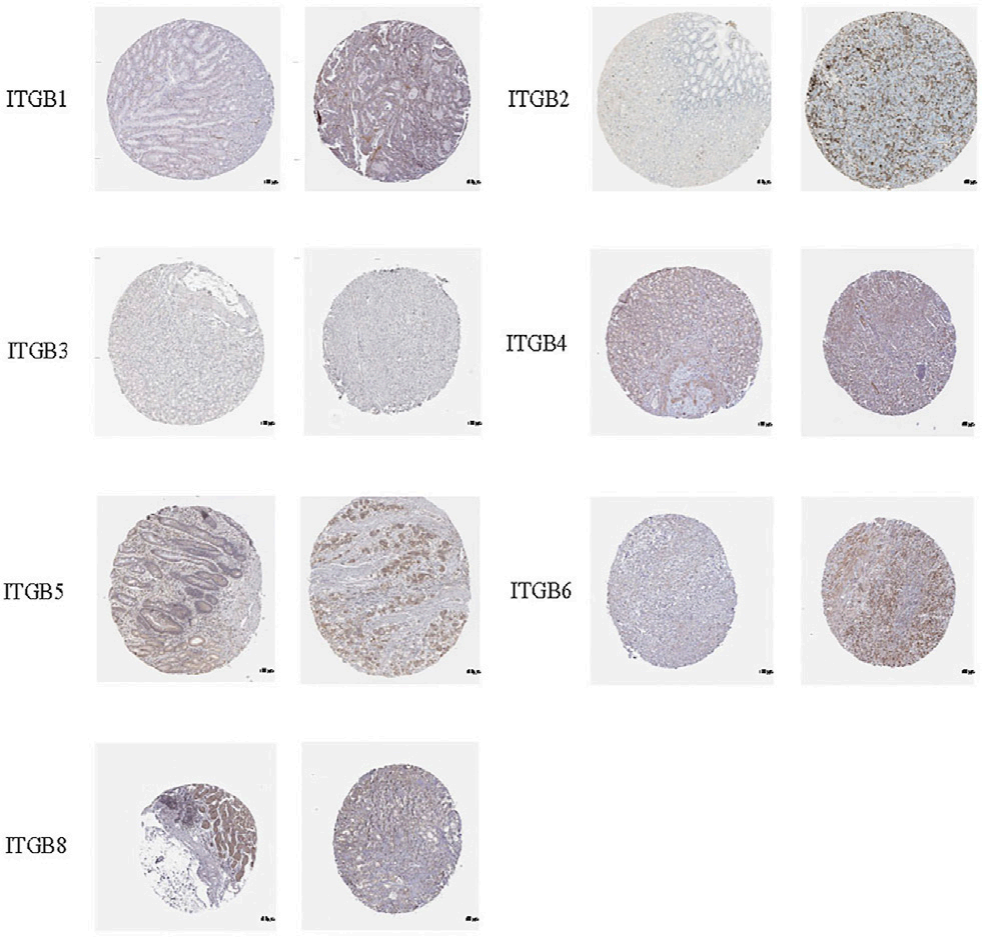
IHC analysis of ITGBs in GC (the HPA database).

IHC was used to examine the protein expression of ITGBs in normal and GC tissues. According to the degree of staining, we found that ITGB1, ITGB2, ITGB4, ITGB5, ITGB6, and ITGB8 proteins were more highly expressed in the GC tissues than in the normal tissues (Figure 4), and these findings were consistent with the mRNA expression. Unfortunately, there is no available protein expression of ITGB7.

### Distinction of Prognostic and Differentially Expressed Genes

**FIGURE 5:**
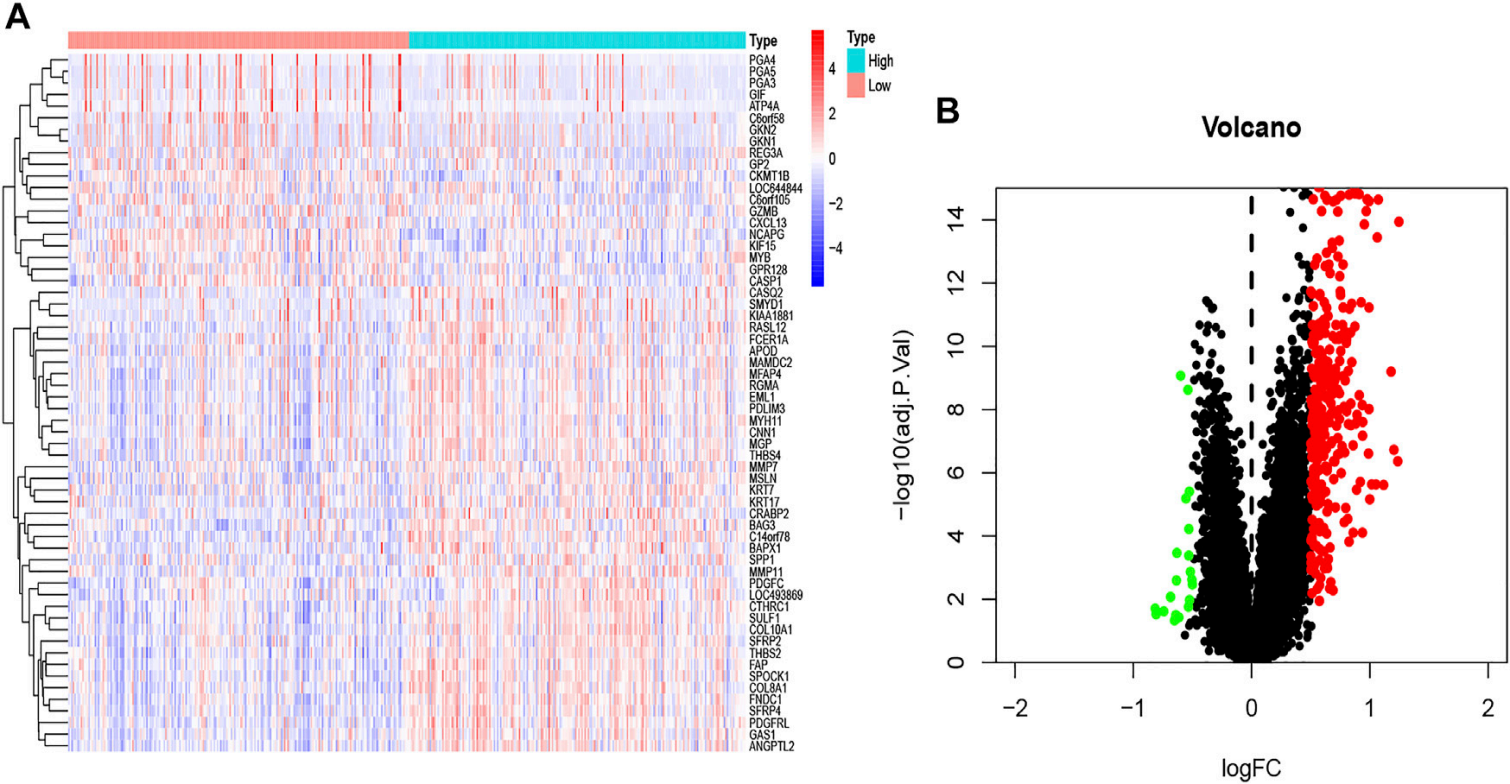
The heatmap **(A)** and volcano plot **(B)** of mRNA expression changes based on ITGB5 in GC samples from the GEO database.

**FIGURE 6:**
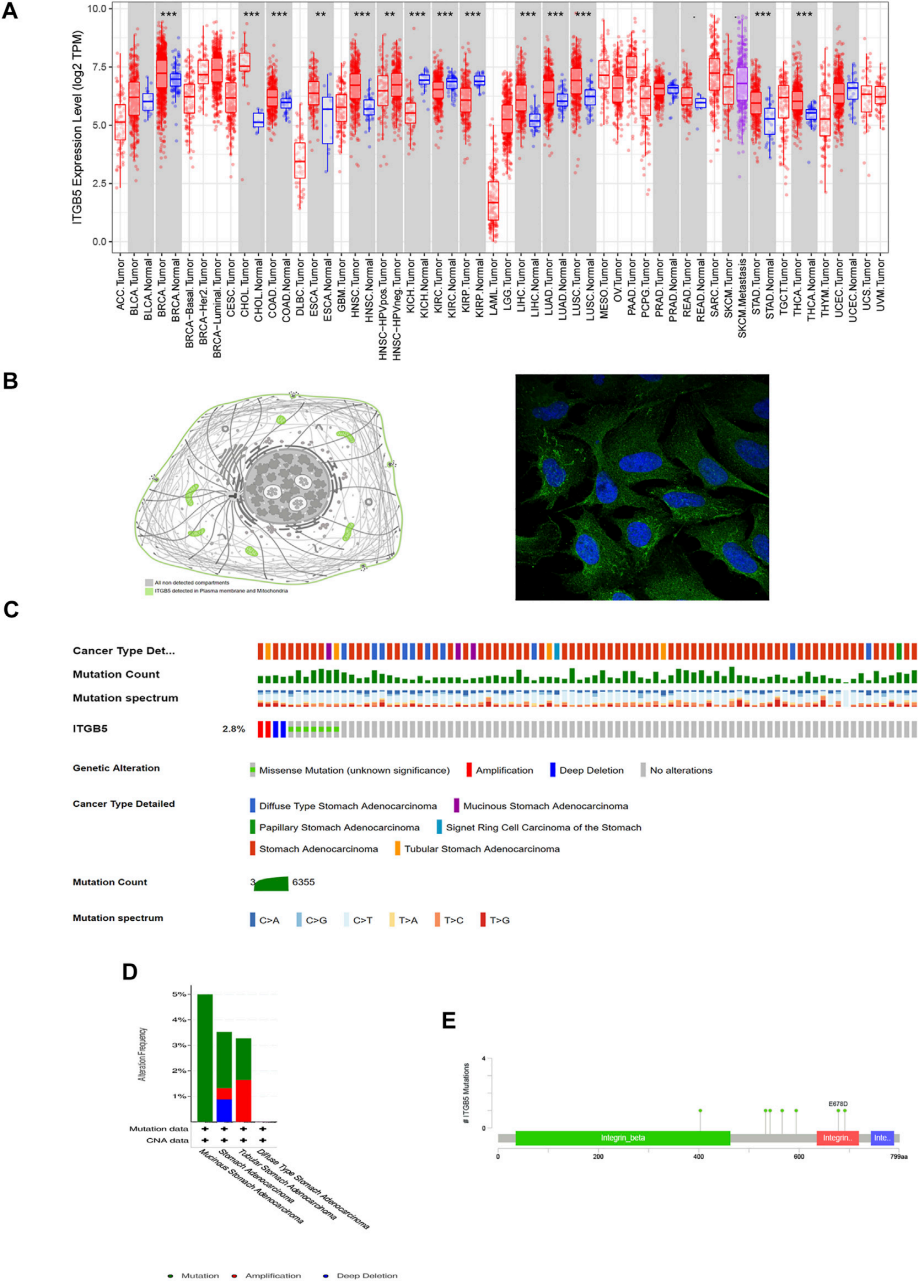
Basic characteristic of ITGB5. **(A)** Expression of ITGB5 in different types of cancer. **(B)** Subcellular location and immunofluorescence images of ITGB5 expression in GC cells from HPA. **(C–E)** Detailed mutation information of ITGB5 in GC.

A total of 172 genes were identified and analyzed for prognosis of GC (Supplementary Table S1), and 334 differentially expressed GC-related genes, which were analyzed from the GEO database, are listed in Supplementary Table S2. ITGB5 exists in both Supplementary Tables S1, S2. Thus, we selected ITGB5 for further analysis. The heatmap and volcano plot show that the ITGB5 interactive genes in GC between the low- and high-ITGB5 expression groups were mainly upregulated genes (Figures 5A,B). The expressions of ITGB5 among various cancer types are shown in Figure 6A as determined by GEPIA. Subcellular location and immunofluorescence images of ITGB5 expression in GC cells were discovered from HPA (Figure 6B). We analyzed the ITGB5 mutation by using the cBioPortal for GC, and the detailed mutation information of ITGB5 in GC is described in Figures 6C–E.

### ITGB5 Expression Predicted Survival and Could Be Used as an Independent Prognostic Biomarker in GC Patients

**FIGURE 7:**
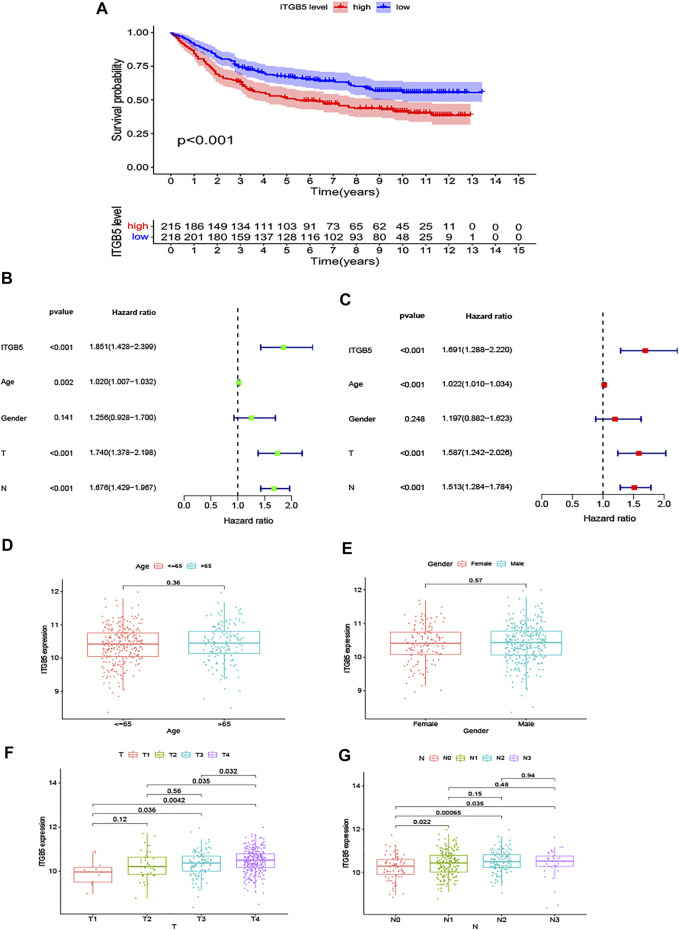
The results in the GEO test cohort. **(A)** The K-M curves for the two groups (high vs. low expression). **(B,C)** The forest maps of univariate and multivariate Cox regression analysis in the GEO data set. **(D,G)** The expression of ITGB5 assigned by clinical factors, comprising age, gender, T stage, and N stage.

**FIGURE 8:**
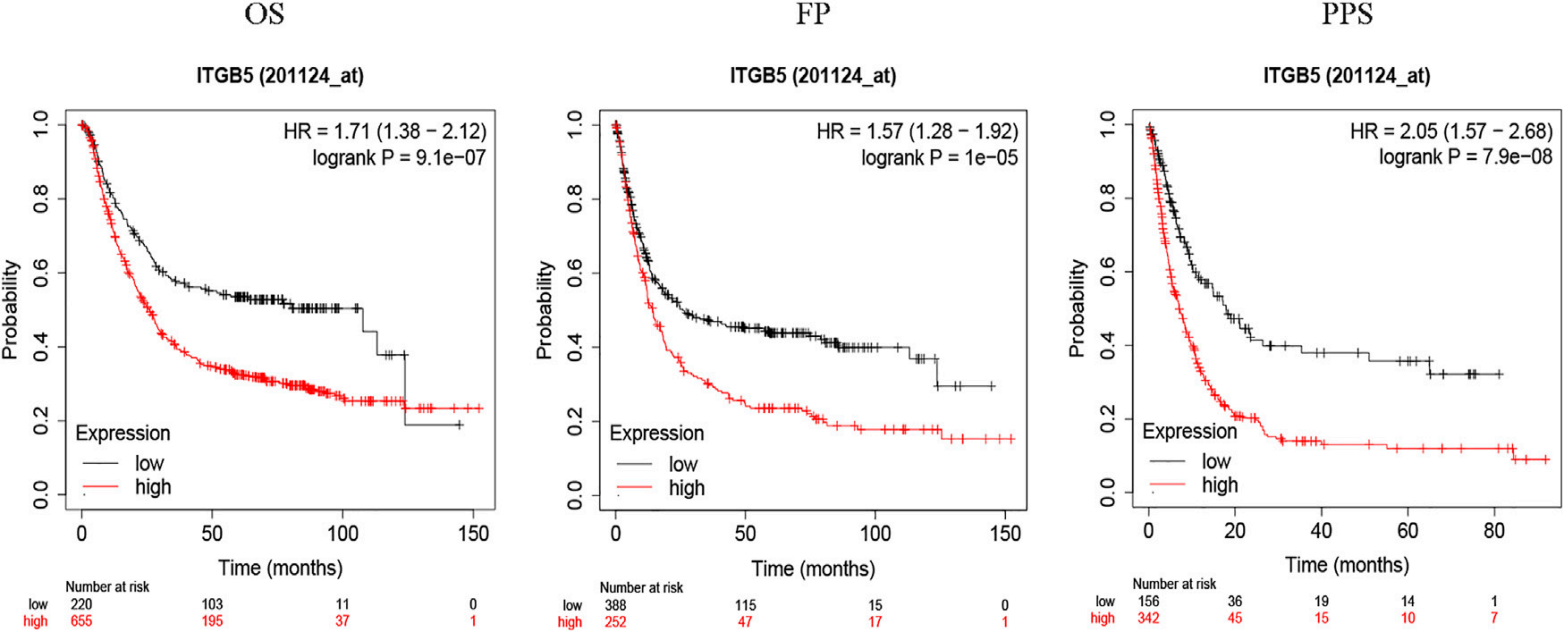
The prognostic value of ITGB5 in GC (Kaplan–Meier Plotter).

To explore the relationship between ITGB5 expression and prognosis, the expression levels of ITGB5 in patients were divided into low- and high-expression groups according to the median value of ITGB5 expression levels in the GEO database. We found that high ITGB5 expression was significantly associated with shorter survival time than those with low ITGB5 expression in GC (Figure 7A). To explore the independence of ITGB5, univariate and multivariate Cox analyses were performed using the entire GEO cohort. The univariate analysis demonstrated that age, stage, and ITGB5 level were independently associated with OS in GC (*p* < .001) (Figure 7B), and the results show that ITGB5 level could be an independent survival predictor of OS in multivariate analyses (*p* < .001) (Figure 7C). Thus, ITGB5 level may serve as an independent predictive and prognostic factor. Conversely, there were significant correlations between the ITGB5 level and T/N stage (Figures 7D–G). The prognostic value of ITGB5 mRNA expression was further verified by using an online database, Kaplan–Meier Plotter (Figure 8). The correlation between ITGB5 expression and various clinical parameters was found by using univariate and multivariable Cox regression. As shown in Figures 7B,C, ITGB5 expression, age, T stage, and N stage are all significantly correlated with OS and are independent prognostic factors. Therefore, ITGB5 could be used as an independent prognostic biomarker in GC patients. Besides this, we performed a nomogram on the foundation of the GEO data set to anticipate the 1-, 2-, and 3-year OS of each GC patient (Supplementary Figure S1A), and the calibration curve of the 3-year OS was obtained, which compared well with the ideal model (Supplementary Figure S1B).

### Functional Enrichment Analysis of the Differentially Expressed Genes in GC Patients

**FIGURE 9:**
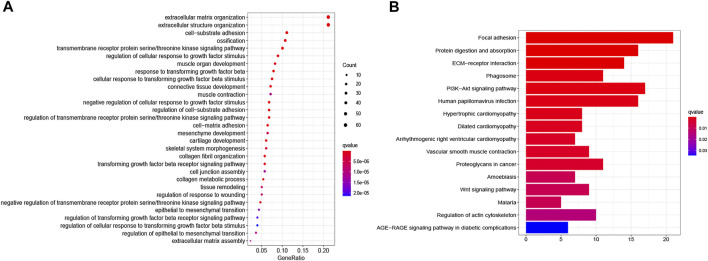
The gene set enrichment analysis. **(A)** Bar plot of GO enrichment. **(B)** Bar plot of KEGG enriched terms.

**FIGURE 10:**
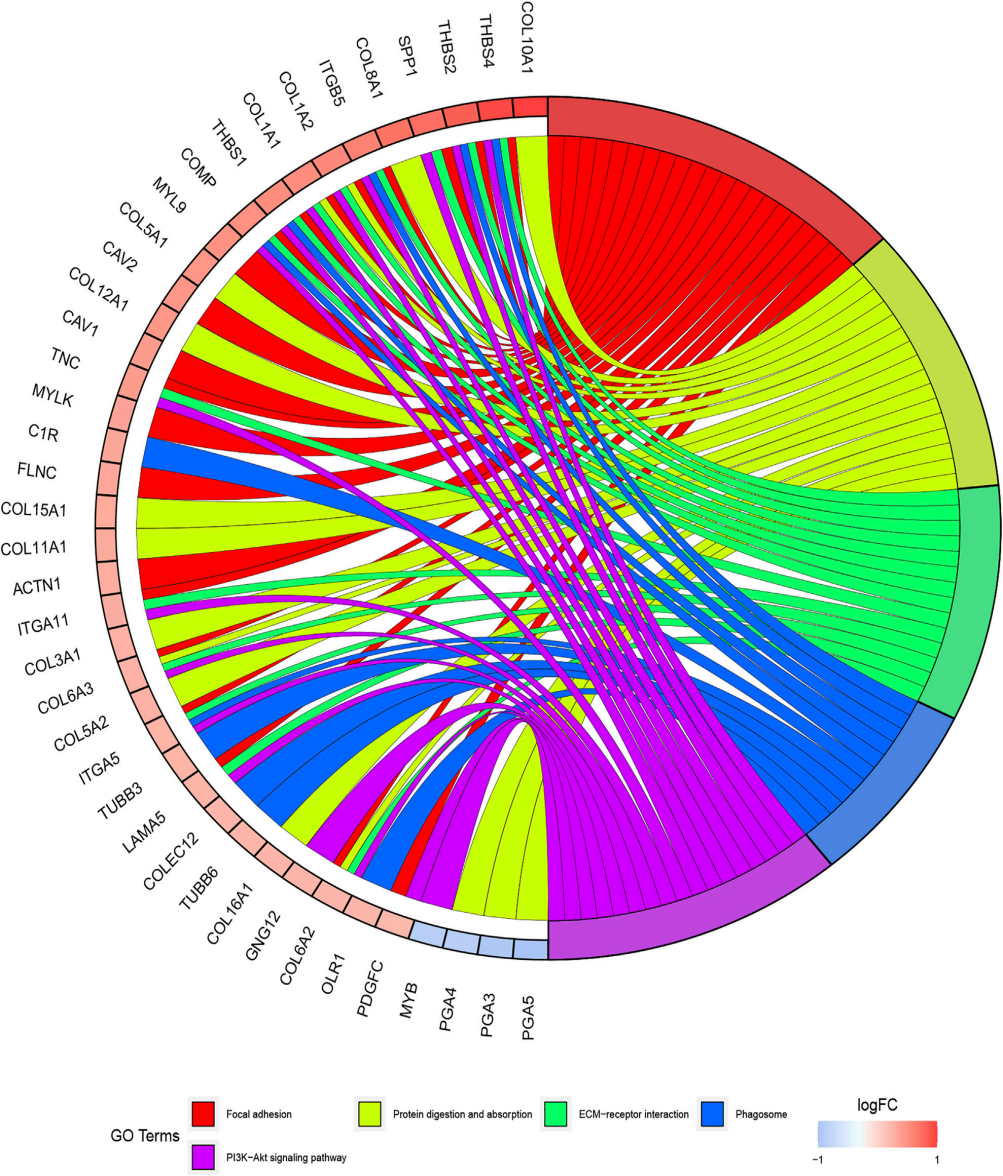
Significant KEGG pathway determined by circos plot.

**FIGURE 11:**
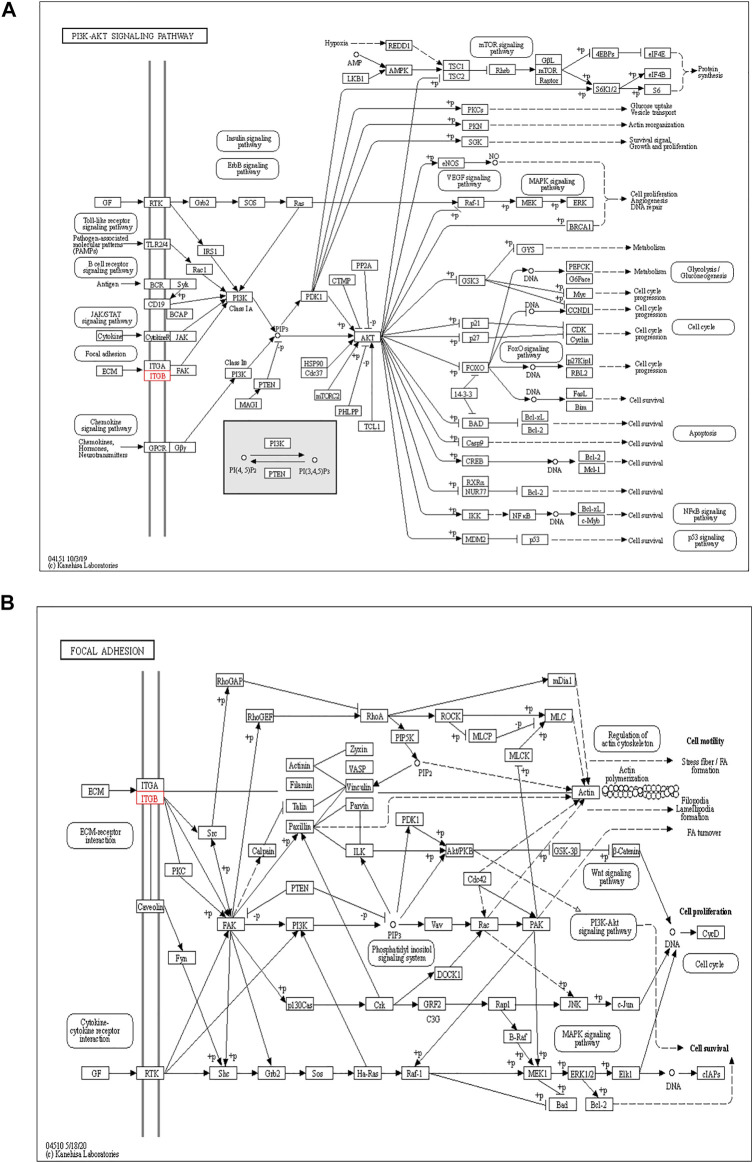
PI3K-Akt signaling pathway **(A)** and focal adhesion procedure **(B)** regulated by ITGBs.

The functions of ITGB5 and the differentially expressed genes significantly associated with GC were predicted by analyzing the GO and KEGG pathway. GO enrichment analysis predicted the functional effect of target genes on account of three aspects, including molecular functions (MF), biological processes (BP), and cellular components (CC). We found that GO:0030198 (extracellular matrix organization), GO:0043062 (extracellular structure organization), GO:0031589 (cell-substrate adhesion), GO:0001503 (ossification), and GO:0007178 (transmembrane receptor protein serine/threonine kinase signaling pathway) were significantly regulated in GC (Figures 9A, 10). In the KEGG pathway analysis, 16 pathways related to the functions of ITGB5 interactive genes were discovered, and the top five pathways identified were focal adhesion, protein digestion and absorption, ECM-receptor interaction, phagosome, and PI3K-Akt signaling pathway (Figure 9B). The PI3K-Akt signaling pathway was involved in the development of GC (Figures 11A,B). These findings indicate that ITGB5 has potential value in the development and metastasis of GC.

## Relationship Between the ITGB5 Expression and the Tumor-Infiltrating Immune Cells

**FIGURE 12:**
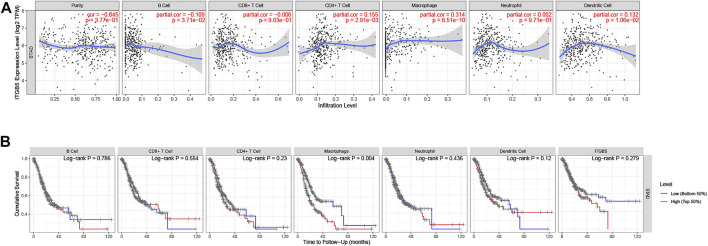
Immune correlation analysis of ITGB5 based on immune infiltration in GC. **(A)** Correlation of ITGB5 expression with immune cell infiltration. **(B)** Prognostic value of immune cell infiltration in GC.

**FIGURE 13:**
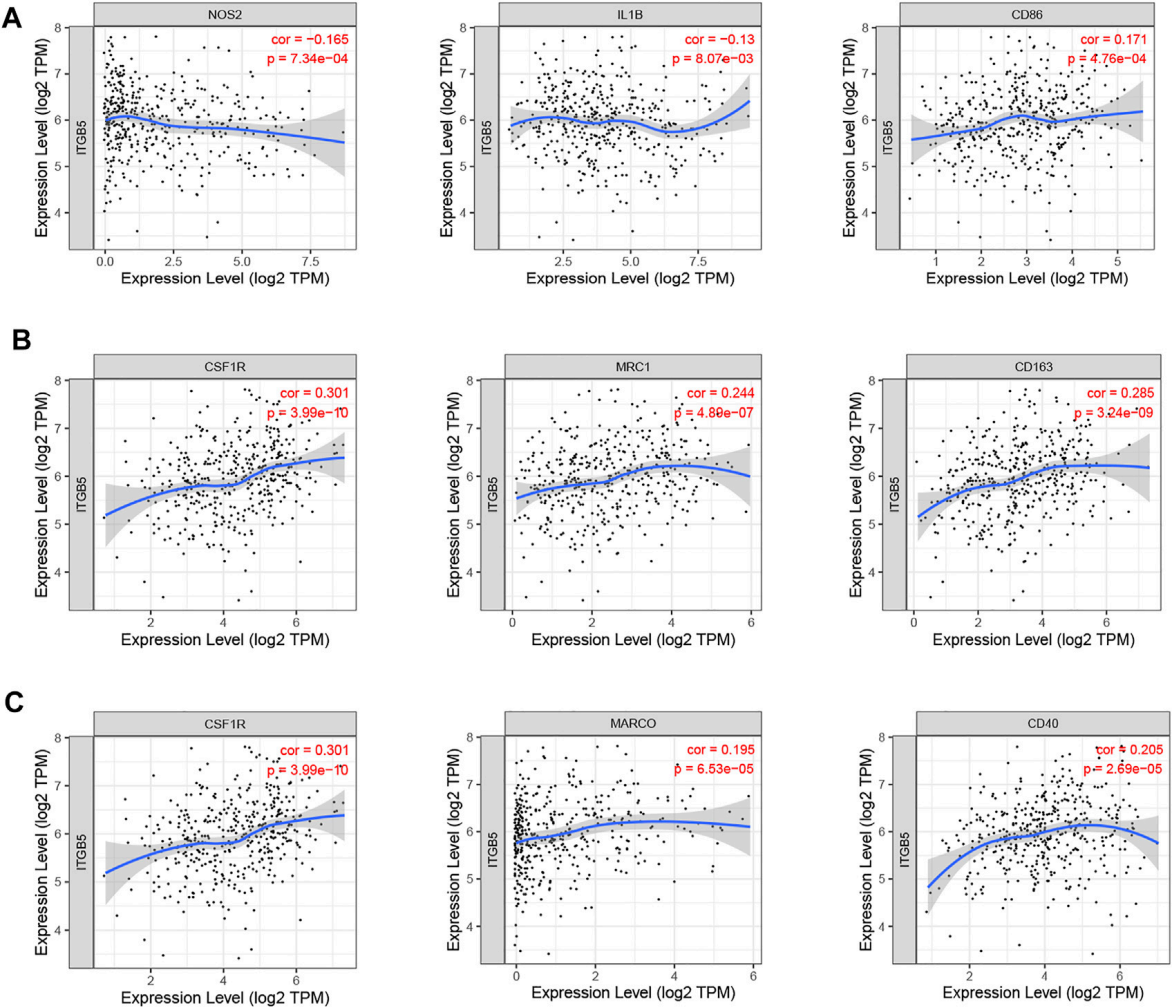
Correlation between ITGB5 expression and gene markers of macrophage in GC. **(A)** ITGB5 and M1 macrophages. **(B)** ITGB5 and M2 macrophages. **(C)** ITGB5 and tumor-associated macrophages.

**FIGURE 14:**
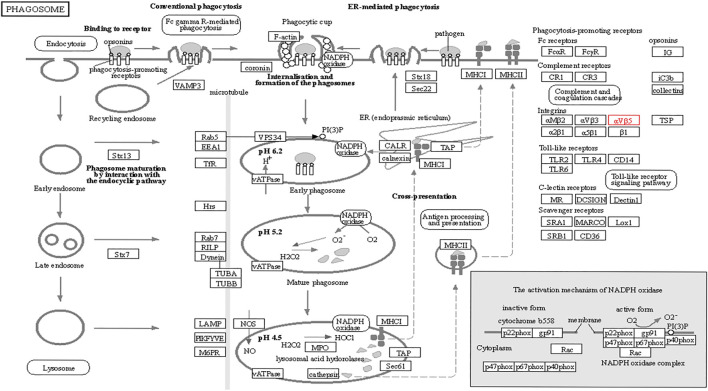
Phagosome pathway regulated by ITGB5.

We used the TIMER algorithm to investigate whether ITGB5 expression is associated with immune infiltration in GC and the abundance of six tumor-infiltrating immune cells (B cells, CD4^+^ T cells, CD8^+^ T cells, macrophages, neutrophils, and dendritic cells). The results shown in Figure 12A indicate that the expression of ITGB5 is positively correlated with CD4^+^ T cells (cor = 0.155, *p* = 2.91e-03), macrophages (cor = 0.314, *p* = 6.51e-10), and dendritic cells (cor = 0.132, *p* = 1.06e-02), whereas it was negatively correlated with B cells (cor = −0.109, *p* = 3.71e-02). The macrophage infiltration significantly correlated with the prognosis of GC patients in KM survival analysis (Figure 12B). Moreover, we plotted the correlation between ITGB5 expression and gene markers of macrophage, and the ITGB5 expression was significantly correlated with macrophage markers, including M1 macrophages markers (NOS2, IL1B, CD86), M2 macrophages markers (CSF1R, MRC1, CD163), and tumor-associated macrophage markers (MARCO, CSF1R, CD40) (Figures 13A–C). The upper findings imply that ITGB5 might involve in infiltration of macrophages and affect patient prognosis *via* regulating immune infiltrates in GC (Figure 14).

## Discussion

Although the past 20 years have been characterized by the expansion of clarifying the molecular mechanism of GC and an advance in diagnostic and therapeutic methods for managing GC patients, the survival outcomes remained poor (Liu et al., 2020; Sugawara et al., 2021). Hence, searching for specific molecular biomarkers for the tumorigenesis and pathogenesis of GC had important significance in the diagnosis and treatment of patients. Since their discovery in the late 1980s, the ITGB superfamily members are demonstrated to be involved in the regulation of cancer development and progression (Cooper and Giancotti, 2019). The role of ITGBs in the tumorigenesis and pathogenesis of various cancer types has increasingly received attention (Sharma et al., 2020; Fujita et al., 2021; Nurzat et al., 2021; Paindelli et al., 2021). Here, in this paper, we evaluate the utility of ITGBs as biomarkers in GC, and we utilize bioinformatics tools for investigating the underlying mechanisms by ITGB5 affected GC.

We explored the mRNA levels of ITGB superfamily members in GC, and all ITGBs except ITGB3 were significantly higher in primary tumors compared with normal tissues in GC. The IHC results show the consistency between RNA and protein expression levels. Furthermore, we investigated correlations between ITGB expression levels and patient prognosis using GEPIA. The results reveal that the expression levels of ITGA1 and ITGB5 were remarkably associated with OS in GC patients. Similar findings were reported in other types of cancers (Yang et al., 2014; Laudato et al., 2017). Xu et al. (2010) established a model that can measure the ITGB5 and ITGB1 expression to predict the survival of GC patients. Taken together, ITGA1 and ITGB5 could be utilized as promising prognostic biomarkers in GC patients. The GEO data set is used as the training and test sets, and we further identified prognosis-related genes. Classified according to GC and normal tissues, we screened differentially expressed GC driver genes. ITGB5 exists in both prognosis-related and differentially expressed genes. Thus, we selected ITGB5 for further analysis. Hirano et al. conducted an *in silico* analysis and found that a high integrin β5 mRNA expression level was correlated with a poor prognosis of patients with GC (Hirano et al., 2020). Similarly, Wang et al. (2020) also suggests that increased ITGB5 level in clinical specimens predicts poor prognosis in GC. We discovered ITGB5 expression upregulated in GC tissues, and the high ITGB5 expression group was positively correlated with advanced tumor stage and positive lymph nodes, which caused a worse prognosis in GC. Our study confirms a high ITGB5 expression level could act as a good predictor of poor prognosis for patients with GC.

However, few studies focus on ITGB5 in GC, and its role in the development and metastasis of GC is not yet fully elucidated (Kawahar et al., 1995; Böger et al., 2015; Lv et al., 2021). ITGB5, encoding integrin-β5, was localized to the plasma membrane and mitochondria, which was supposed to be associated with the initiation and progression of the tumor by mediating links between the ECM and cells (Zhang et al., 2019). According to the enrichment of GO collection, we found that ITGB5 expression is significantly associated with ECM organization, extracellular structure organization, cell-substrate adhesion, and ossification. Exploring the molecular mechanisms of ITGB5 in GC contributed to investigating the novel targeted therapy approach. The KEGG pathway analysis of ITGB5 shows a close association between ITGB5 and focal adhesion, protein digestion and absorption, ECM-receptor interaction, phagosome, and PI3K-Akt signaling pathway. Several studies already report that ITGBs mediate the interaction between ECM and cells and are involved in cell adhesion and migration (Wilisch-Neumann et al., 2013; Lian et al., 2016). Cell adhesion molecules act as one of the main mediators between the ECM and cell. Changes in cell adhesion molecules may affect a variety of signaling pathways, leading to the occurrence and development of tumors (Deville and Cordes, 2019). Previous findings indicate that the focal adhesion signaling pathway plays an important role in the process of epithelial to mesenchymal transition in pancreatic cancer (Ning et al., 2014). Wang et al. (2016a) reports that the focal adhesion signaling pathway activated by ITGB5 can alter cell glycolysis and induce cisplatin resistance in cervical and breast cancer. Meanwhile, for the first time, we demonstrate that ITGB5 overexpression was significantly associated with upregulation of the focal adhesion signaling pathway, indicating the potential role of ITGB5 in the focal adhesion signaling pathway in GC. We also found that the PI3K-Akt signaling pathway is upregulated. The anomalous activation of the PI3K/Akt signal pathway presents in variable tumors, and plenty of studies verify that the PI3K-Akt signal pathway is involved in regulating GC cell growth, proliferation, migration, differentiation, apoptosis, and energy metabolism (Lin et al., 2019; Rong et al., 2020; Xu et al., 2020). However, the research about its mechanism through ITGB5 in GC is insufficient, and there is no direct evidence to confirm that the upregulation of this pathway affiliates with the prognosis of GC. Indeed, the results suggest that we need further work on the relationship between the ITGB5 and PI3K-Akt in GC.

Cellular immunity plays a considerable role in the antitumor process. B cells, T cells, macrophages, neutrophils, and dendritic cells are the main effector cells of the immune system. The infiltrating condition of these immune cells and their differences in forecasting the prognosis of GC has not been discussed yet. To further study the function of ITGB5 in GC, we analyzed whether ITGB5 expression is associated with immune infiltration by using the TIMER algorithm, and the results show that the content of these special immune cells activated was statistically different. In our paper, we find the infiltrating level of CD4^+^ T cells, macrophages, and dendritic cells were positively related to the expression of ITGB5, especially macrophages, and a low level of macrophages predicts a better prognosis in GC in our study. Besides this, we explored the correlation between ITGB5 expression and gene markers of macrophage, and the ITGB5 expression was significantly correlated with macrophage markers, including M1 macrophage markers (NOS2, IL1B, CD86), M2 macrophage markers (CSF1R, MRC1, CD163), and tumor-associated macrophage markers (MARCO, CSF1R, CD40). Macrophages as a fundamental innate immune population perform various supportive functions specialized to different tissue components, and aberrance in macrophage functions leads substantially to the development and progression of several diseases, including cancer (Ngambenjawong et al., 2017). Tumor-associated macrophages, M1 and M2 macrophage phenotypes, are generally considered to directly or indirectly promote tumor proliferation and metastasis in GC and are positively correlated with invasion depth and tumor stage (Ishigami et al., 2003). The M1 macrophages can stimulate apoptosis, suppress proliferation and the development of neovascularization, whereas M2 macrophages can accelerate both cancer growth and metastasis (Wu et al., 2015; Wang et al., 2016b). In recent years, researchers have expanded their studies to figure out both M1 and M2 phenotypes within microenvironments. High levels of M1 macrophages predict better prognosis, whereas increasing levels of M2 macrophages indicate poor outcomes (Ma et al., 2010; Yuan et al., 2014; Mei et al., 2016). Liu et al. (2020) summarize that the high infiltrating levels of M2 macrophages and total tumor-associated macrophages might be negative prognostic factors for patients with GC. In our present study, M2 macrophages were positively and M1 macrophages negatively related to the expression of ITGB5. The total tumor-associated macrophages were positively correlated with the level of ITGB5, which indicates higher M2 macrophage infiltration, thus supporting the view that ITGB5 may play a vital role in the progression of GC via promoting M2 macrophage polarization and inhibiting antitumor immunity. This might explain, to a certain degree, why GC patients with high expression of ITGB5 had poor prognoses. Along with the existing evidence, the results of our study confirm that ITGB5 might act a critical role in the immune mechanism of cancer.

To the best of our knowledge, this is the first study to comprehensively identify the oncologic and prognostic values of the eight ITGBs. The upper results demonstrate that ITGB5 has potential value in the tumorigenesis and pathogenesis of GC, which might involve infiltration of macrophages and affect patient prognosis *via* regulating immune infiltrates in GC. However, this study has some limitations that should be considered. On the one hand, our study is based on the specimens from one database and may have a bias by the potential heterogeneity. On the other hand, this study was only at the level of bioinformatics analysis, and *in vitro* and *in vivo* experiments about the underlying mechanism of ITGB5 in GC should be performed.

In conclusion, ITGB5 seems to be a valuable prognostic biomarker and a pivotal regulator of immune infiltrates in GC patients, which might be a potential target of precision therapy against GC. However, the molecular mechanism and the related signal pathways of ITGB5 in GC remain unclear, which requires further exploration.

## Data Availability

The original contributions presented in the study are included in the article/Supplementary Material, further inquiries can be directed to the corresponding authors.

## References

[B1] An X.-Z. Zhao Z.-G. Luo Y.-X. Zhang R. Tang X.-Q. Hao D.-L. (2016). Netrin-1 Suppresses the MEK/ERK Pathway and ITGB4 in Pancreatic Cancer. Oncotarget. 7 (17), 24719–24733. 10.18632/oncotarget.8348 27034160 PMC5029736

[B2] Bianconi D. Unseld M. Prager G. W. (2016). Integrins in the Spotlight of Cancer. Int. J. Mol. Sci. 17 (12), 2037. 10.3390/ijms17122037 PMC5187837 27929432

[B3] Böger C. Warneke V. S. Behrens H.-M. Kalthoff H. Goodman S. L. Becker T. (2015). Integrins αvβ3 and αvβ5 as Prognostic, Diagnostic, and Therapeutic Targets in Gastric Cancer. Gastric Cancer. 18 (4), 784–795. 10.1007/s10120-014-0435-2 25315085 PMC4572058

[B4] Chung J. Kim T. H. (2008). Integrin-Dependent Translational Control: Implication in Cancer Progression. Microsc. Res. Tech. 71 (5), 380–386. 10.1002/jemt.20566 18300291

[B5] Compton C. C. (2007). Optimal Pathologic Staging: Defining Stage II Disease. Clin. Cancer Res. 13 (22 Pt 2), 6862s–70s. 10.1158/1078-0432.CCR-07-1398 18006791

[B6] Cooper J. Giancotti F. G. (2019). Integrin Signaling in Cancer: Mechanotransduction, Stemness, Epithelial Plasticity, and Therapeutic Resistance. Cancer Cell. 35 (3), 347–367. 10.1016/j.ccell.2019.01.007 30889378 PMC6684107

[B7] Cui Y. Wu F. Tian D. Wang T. Lu T. Huang X. (2018). miR-199a-3p Enhances Cisplatin Sensitivity of Ovarian Cancer Cells by Targeting ITGB8. Oncol. Rep. 39 (4), 1649–1657. 10.3892/or.2018.6259 29436681 PMC5868401

[B8] Deville S. S. Cordes N. (2019). The Extracellular, Cellular, and Nuclear Stiffness, a Trinity in the Cancer Resistome-A Review. Front. Oncol. 9, 1376. 10.3389/fonc.2019.01376 31867279 PMC6908495

[B9] Ferlay J. Colombet M. Soerjomataram I. Mathers C. Parkin D. M. Piñeros M. (2019). Estimating the Global Cancer Incidence and Mortality in 2018: GLOBOCAN Sources and Methods. Int. J. Cancer. 144 (8), 1941–1953. 10.1002/ijc.31937 30350310

[B10] Fujita M. Sasada M. Eguchi M. Iyoda T. Okuyama S. Osawa T. (2021). Induction of Cellular Senescence in Fibroblasts through β1-integrin Activation by Tenascin-C-Derived Peptide and its Protumor Effect. Am. J. Cancer Res. 11 (9), 4364–4379. 34659892 PMC8493383

[B11] Ginsberg M. H. (2014). Integrin Activation. BMB Rep. 47 (12), 655–659. 10.5483/bmbrep.2014.47.12.241 25388208 PMC4345508

[B12] Hirano T. Shinsato Y. Tanabe K. Higa N. Kamil M. Kawahara K. (2020). FARP1 Boosts CDC42 Activity from Integrin αvβ5 Signaling and Correlates with Poor Prognosis of Advanced Gastric Cancer. Oncogenesis. 9 (2), 13. 10.1038/s41389-020-0190-7 32029704 PMC7005035

[B13] Huang L. Hu C. Chao H. Wang R. Lu H. Li H. (2019). miR-29c Regulates Resistance to Paclitaxel in Nasopharyngeal Cancer by Targeting ITGB1. Exp. Cell Res. 378 (1), 1–10. 10.1016/j.yexcr.2019.02.012 30779921

[B14] Ilhan E. Ureyen O. Meral U. M. (2016). Ongoing Problems Concerning 7th TNM Staging System and Proposals for 8th TNM Staging System of Gastric Cancer. pg. 4 (4), 223–225. 10.5114/pg.2016.64069 PMC5209469 28053675

[B15] Ishigami S. Natsugoe S. Tokuda K. Nakajo A. Okumura H. Matsumoto M. (2003). Tumor-Associated Macrophage (TAM) Infiltration in Gastric Cancer. Anticancer Res. 23 (5A), 4079–4083. 14666722

[B16] Kawahar E. Ooi A. Nakanishi I. (1995). Integrin Distribution in Gastric Carcinoma: Association of β3 and β5 Integrins with Tumor Invasiveness. Pathol. Int. 45 (7), 493–500. 10.1111/j.1440-1827.1995.tb03491.x 7551009

[B17] Laudato S. Patil N. Abba M. L. Leupold J. H. Benner A. Gaiser T. (2017). P53-induced miR-30e-5p Inhibits Colorectal Cancer Invasion and Metastasis by Targeting ITGA6 and ITGB1. Int. J. Cancer. 141 (9), 1879–1890. 10.1002/ijc.30854 28656629

[B18] Lian P.-L. Liu Z. Yang G.-Y. Zhao R. Zhang Z.-Y. Chen Y.-G. (2016). Integrin αvβ6 and Matrix Metalloproteinase 9 Correlate with Survival in Gastric Cancer. Wjg. 22 (14), 3852–3859. 10.3748/wjg.v22.i14.3852 27076771 PMC4814749

[B19] Lin J.-X. Xie X.-S. Weng X.-F. Qiu S.-L. Yoon C. Lian N.-Z. (2019). UFM1 Suppresses Invasive Activities of Gastric Cancer Cells by Attenuating the Expression of PDK1 through PI3K/AKT Signaling. J. Exp. Clin. Cancer Res. 38 (1), 410. 10.1186/s13046-019-1416-4 31533855 PMC6751655

[B20] Lin Z. He R. Luo H. Lu C. Ning Z. Wu Y. (2018). Integrin-β5, a miR-185-Targeted Gene, Promotes Hepatocellular Carcinoma Tumorigenesis by Regulating β-catenin Stability. J. Exp. Clin. Cancer Res. 37 (1), 17. 10.1186/s13046-018-0691-9 29386044 PMC5793391

[B21] Liu X. Cao Y. Li R. Gu Y. Chen Y. Qi Y. (2020). Poor Clinical Outcomes of Intratumoral Dendritic Cell-Specific Intercellular Adhesion Molecule 3-grabbing Non-integrin-positive Macrophages Associated with Immune Evasion in Gastric Cancer. Eur. J. Cancer. 128, 27–37. 10.1016/j.ejca.2020.01.002 32109848

[B22] Lv Y. Shan Y. Song L. Zhao Y. Lai R. Su J. (2021). Type I Collagen Promotes Tumor Progression of Integrin β1 Positive Gastric Cancer through a BCL9L/β-Catenin Signaling Pathway. Aging. 13 (14), 19064–19076. 10.18632/aging.203355 34319913 PMC8351671

[B23] Ma J. Liu L. Che G. Yu N. Dai F. You Z. (2010). The M1 Form of Tumor-Associated Macrophages in Non-Small Cell Lung Cancer Is Positively Associated with Survival Time. BMC Cancer 10, 112. 10.1186/1471-2407-10-112 20338029 PMC2851690

[B24] Mei J. Xiao Z. Guo C. Pu Q. Ma L. Liu C. (2016). Prognostic Impact of Tumor-Associated Macrophage Infiltration in Non-small Cell Lung Cancer: A Systemic Review and Meta-Analysis. Oncotarget. 7 (23), 34217–34228. 10.18632/oncotarget.9079 27144518 PMC5085150

[B25] Ngambenjawong C. Gustafson H. H. Pun S. H. (2017). Progress in Tumor-Associated Macrophage (TAM)-Targeted Therapeutics. Adv. Drug Deliv. Rev. 114, 206–221. 10.1016/j.addr.2017.04.010 28449873 PMC5581987

[B26] Ning Z. Wang A. Liang J. Xie Y. Liu J. Yan Q. (2014). USP22 Promotes Epithelial-Mesenchymal Transition via the FAK Pathway in Pancreatic Cancer Cells. Oncol. Rep. 32 (4), 1451–1458. 10.3892/or.2014.3354 25070659

[B27] Nurzat Y. Su W. Min P. Li K. Xu H. Zhang Y. (2021). Identification of Therapeutic Targets and Prognostic Biomarkers Among Integrin Subunits in the Skin Cutaneous Melanoma Microenvironment. Front. Oncol. 11, 751875. 10.3389/fonc.2021.751875 34660316 PMC8514842

[B28] Paindelli C. Casarin S. Wang F. (2021). Enhancing Radium 223 Treatment Efficacy by Anti-beta 1 Integrin Targeting. J. Nucl. Med. 10.2967/jnumed.121.262743 PMC9258579 34711616

[B29] Rong L. Li Z. Leng X. Li H. Ma Y. Chen Y. (2020). Salidroside Induces Apoptosis and Protective Autophagy in Human Gastric Cancer AGS Cells through the PI3K/Akt/mTOR Pathway. Biomed. Pharmacother. 122, 109726. 10.1016/j.biopha.2019.109726 31918283

[B30] Sharma R. Kameswaran M. Dash A. (2020). ComparativeIn VitroCytotoxicity Studies of177Lu-CHX-A″-DTPA-Trastuzumab and177Lu-CHX-A″-DTPA-F(ab′)2-Trastuzumab in HER2-Positive Cancer Cell Lines. Cancer Biother. Radiopharm. 35 (3), 177–189. 10.1089/cbr.2019.2882 32196365

[B31] Shen J. Xu J. Chen B. Ma D. Chen Z. Li J.-C. (2019). Elevated Integrin α6 Expression Is Involved in the Occurrence and Development of Lung Adenocarcinoma, and Predicts a Poor Prognosis: a Study Based on Immunohistochemical Analysis and Bioinformatics. J. Cancer Res. Clin. Oncol. 145 (7), 1681–1693. 10.1007/s00432-019-02907-1 31175464 PMC11810158

[B32] Song J. Yang P. Lu J. (2020). Upregulation of ITGBL1 Predicts Poor Prognosis and Promotes Chemoresistance in Ovarian Cancer. Cancer Biomark. 27 (1), 51–61. 10.3233/CBM-190460 31683459 PMC12662264

[B33] Sugawara K. Yamashita H. Yajima S. Oshima Y. Mitsumori N. Fujisaki M. (2021). Prognosis of Hemodialysis Patients Undergoing Surgery for Gastric Cancer: Results of a Multicenter Retrospective Study. Surgery. 170 (1), 249–256. 10.1016/j.surg.2021.01.014 33632543

[B34] Wang J. F. Wang Y. Zhang S. W. Chen Y. Y. Qiu Y. Duan S. Y. (2020). Expression and Prognostic Analysis of Integrins in Gastric Cancer. J. Oncol. 2020, 8862228. 10.1155/2020/8862228 33335550 PMC7722456

[B35] Wang S. Xie J. Li J. Liu F. Wu X. Wang Z. (2016a). Cisplatin Suppresses the Growth and Proliferation of Breast and Cervical Cancer Cell Lines by Inhibiting Integrin β5-mediated Glycolysis. Am. J. Cancer Res. 6 (5), 1108–1117. 27294003 PMC4889724

[B36] Wang X. L. Jiang J. T. Wu C. P. (2016b). Prognostic Significance of Tumor-Associated Macrophage Infiltration in Gastric Cancer: a Meta-Analysis. Genet. Mol. Res. 15 (4), gmr15049040. 10.4238/gmr15049040 27966749

[B37] Wilisch-Neumann A. Kliese N. Pachow D. Schneider T. Warnke J.-P. Braunsdorf W. E. (2013). The Integrin Inhibitor Cilengitide Affects Meningioma Cell Motility and Invasion. Clin. Cancer Res. 19 (19), 5402–5412. 10.1158/1078-0432.ccr-12-0299 23948974

[B38] Wu M.-H. Lee W.-J. Hua K.-T. Kuo M.-L. Lin M.-T. (2015). Macrophage Infiltration Induces Gastric Cancer Invasiveness by Activating the β-Catenin Pathway. PLoS One. 10 (7), e0134122. 10.1371/journal.pone.0134122 26226629 PMC4520459

[B39] Xu L. Chen J. Jia L. Chen X. Awaleh Moumin F. Cai J. (2020). SLC1A3 Promotes Gastric Cancer Progression via the PI3K/AKT Signalling Pathway. J. Cell. Mol. Med. 24 (24), 14392–14404. 10.1111/jcmm.16060 33145952 PMC7753768

[B40] Xu Z.-Y. Chen J.-S. Shu Y.-Q. (2010). Gene Expression Profile Towards the Prediction of Patient Survival of Gastric Cancer. Biomed. Pharmacother. 64 (2), 133–139. 10.1016/j.biopha.2009.06.021 20005068

[B41] Yang Z. Zhou X. Liu Y. Gong C. Wei X. Zhang T. (2014). Activation of Integrin β1 Mediates the Increased Malignant Potential of Ovarian Cancer Cells Exerted by Inflammatory Cytokines. Acamc. 14 (7), 955–962. 10.2174/1871520614666140613123108 24931361

[B42] Yuan Z.-Y. Luo R.-Z. Peng R.-J. Wang S.-S. Xue C. (2014). High Infiltration of Tumor-Associated Macrophages in Triple-Negative Breast Cancer Is Associated with a Higher Risk of Distant Metastasis. Ott. 7, 1475–1480. 10.2147/ott.s61838 PMC4149399 25187727

[B43] Zhang L.-y. Guo Q. Guan G.-f. Cheng W. Cheng P. Wu A.-h. (2019). Integrin Beta 5 Is a Prognostic Biomarker and Potential Therapeutic Target in Glioblastoma. Front. Oncol. 9, 904. 10.3389/fonc.2019.00904 31616629 PMC6764112

