# Supplementary material for: Comprehensive Analysis of the Expression and Prognosis for ITGBs: Identification of ITGB5 as a Biomarker of Poor Prognosis and Correlated with Immune Infiltrates in Gastric Cancer
Source: Front Cell Dev Biol. 2022 Feb 9;9:816230. doi: 10.3389/fcell.2021.816230 (PMC8863963; doi:10.3389/fcell.2021.816230)
Supplement: Supplementary file 1 [file Table1.DOCX]

Supplementary Table 1 Prognostic GC-related genes

| gene | KM | HR | HR.95L | HR.95H | coxPvalue |
| --- | --- | --- | --- | --- | --- |
| ABLIM3 | 0.000436 | 1.515774 | 1.217995 | 1.886355 | 0.000194 |
| ACTG2 | 1.62E-06 | 1.205065 | 1.110931 | 1.307175 | 6.96E-06 |
| AGTR1 | 0.000254 | 1.31356 | 1.119115 | 1.54179 | 0.000848 |
| AKAP14 | 0.000225 | 0.284489 | 0.140597 | 0.575647 | 0.000473 |
| ARHGAP23 | 3.21E-05 | 1.530645 | 1.315111 | 1.781503 | 3.85E-08 |
| BARX1 | 0.000722 | 1.190991 | 1.074656 | 1.319921 | 0.000859 |
| C10orf10 | 0.000228 | 1.350914 | 1.183631 | 1.541839 | 8.22E-06 |
| C10orf56 | 0.000563 | 1.25933 | 1.11267 | 1.425322 | 0.000262 |
| C14orf145 | 0.000569 | 0.534292 | 0.396126 | 0.720647 | 4.03E-05 |
| C14orf78 | 0.00083 | 1.274964 | 1.141802 | 1.423656 | 1.59E-05 |
| C15orf52 | 0.000586 | 1.235979 | 1.10367 | 1.384149 | 0.000245 |
| C1QTNF5 | 0.000188 | 1.303991 | 1.138024 | 1.494162 | 0.000133 |
| C21orf34 | 0.000846 | 1.429582 | 1.190269 | 1.717011 | 0.000132 |
| C2orf40 | 3.06E-05 | 1.124812 | 1.057953 | 1.195897 | 0.000169 |
| C4B | 0.000454 | 3.758302 | 1.993808 | 7.084348 | 4.25E-05 |
| C5orf20 | 0.000658 | 0.444456 | 0.301963 | 0.65419 | 3.93E-05 |
| C7orf10 | 2.31E-05 | 1.384259 | 1.179032 | 1.62521 | 7.14E-05 |
| CALD1 | 4.72E-05 | 1.278128 | 1.137462 | 1.43619 | 3.71E-05 |
| CAP2 | 3.06E-06 | 1.246688 | 1.118228 | 1.389905 | 7.07E-05 |
| CAV1 | 0.000589 | 1.225865 | 1.093711 | 1.373988 | 0.000467 |
| CBLN2 | 4.76E-05 | 1.504346 | 1.227801 | 1.843179 | 8.14E-05 |
| CCDC46 | 0.000786 | 1.591423 | 1.25328 | 2.020798 | 0.000138 |
| CCNJ | 0.000349 | 0.560667 | 0.403379 | 0.779284 | 0.000572 |
| CD160 | 0.000772 | 0.454549 | 0.319161 | 0.64737 | 1.24E-05 |
| CD38 | 0.000116 | 0.727088 | 0.629732 | 0.839495 | 1.39E-05 |
| CDH26 | 7.78E-05 | 2.119146 | 1.383612 | 3.245694 | 0.000555 |
| CHAF1A | 2.57E-05 | 0.622518 | 0.470525 | 0.823608 | 0.000904 |
| CHRDL2 | 1.85E-05 | 1.201199 | 1.087834 | 1.326378 | 0.00029 |
| CHRNA3 | 7.62E-05 | 1.229931 | 1.091198 | 1.386301 | 0.000701 |
| CHST3 | 3.07E-05 | 1.516545 | 1.280417 | 1.796218 | 1.42E-06 |
| CISH | 0.000492 | 0.525491 | 0.388687 | 0.710445 | 2.89E-05 |
| CLEC12A | 0.00065 | 0.470208 | 0.319758 | 0.691447 | 0.000125 |
| CNN1 | 6.81E-07 | 1.15034 | 1.083762 | 1.221009 | 4.14E-06 |
| CNOT6L | 0.000541 | 0.416198 | 0.274146 | 0.631856 | 3.87E-05 |
| COL18A1 | 7.67E-06 | 1.56114 | 1.292538 | 1.885561 | 3.77E-06 |
| COL4A2 | 0.000836 | 1.308858 | 1.123744 | 1.524465 | 0.000541 |
| COL8A2 | 0.000122 | 1.4041 | 1.160643 | 1.698624 | 0.000477 |
| CPXM2 | 1.40E-06 | 1.173085 | 1.069111 | 1.28717 | 0.000748 |
| CRABP2 | 0.000187 | 1.263784 | 1.147279 | 1.392119 | 2.09E-06 |
| CRIP2 | 0.000114 | 1.355708 | 1.18181 | 1.555193 | 1.39E-05 |
| CRYAB | 5.64E-07 | 1.231773 | 1.126679 | 1.34667 | 4.62E-06 |
| CSRP1 | 4.72E-06 | 1.290872 | 1.143601 | 1.457108 | 3.61E-05 |
| CST2 | 0.000331 | 1.346383 | 1.152263 | 1.573205 | 0.000181 |
| CST6 | 0.000576 | 1.267016 | 1.143369 | 1.404035 | 6.27E-06 |
| DACT1 | 2.00E-05 | 1.39012 | 1.168359 | 1.653971 | 0.000203 |
| DACT3 | 7.83E-05 | 1.229176 | 1.114159 | 1.356066 | 3.85E-05 |
| DES | 2.21E-07 | 1.146366 | 1.082953 | 1.213491 | 2.54E-06 |
| DKK3 | 3.48E-05 | 1.366963 | 1.164894 | 1.604085 | 0.000128 |
| DMN | 1.00E-06 | 1.179828 | 1.095445 | 1.27071 | 1.26E-05 |
| DPYSL3 | 0.0003 | 1.217489 | 1.095018 | 1.353657 | 0.000275 |
| FAM26A | 0.000196 | 1.428727 | 1.212507 | 1.683506 | 2.03E-05 |
| FAM43B | 0.000499 | 1.778547 | 1.308035 | 2.418308 | 0.00024 |
| FAM46B | 0.000428 | 1.245946 | 1.115197 | 1.392026 | 0.000101 |
| FEZ1 | 0.000454 | 1.344271 | 1.134905 | 1.59226 | 0.000615 |
| FHL1 | 6.40E-05 | 1.17375 | 1.081471 | 1.273903 | 0.000126 |
| FLJ40448 | 1.39E-05 | 0.408809 | 0.258714 | 0.645983 | 0.000127 |
| FLOT1 | 0.000106 | 1.653498 | 1.295314 | 2.110729 | 5.41E-05 |
| FNDC4 | 0.000634 | 1.564424 | 1.239421 | 1.974649 | 0.000166 |
| FRMD6 | 0.000252 | 1.329818 | 1.133146 | 1.560624 | 0.000482 |
| FZD4 | 0.000225 | 1.41042 | 1.18466 | 1.679203 | 0.000112 |
| GLS2 | 1.01E-05 | 0.474583 | 0.330191 | 0.682116 | 5.65E-05 |
| GREM1 | 0.000289 | 1.219629 | 1.107852 | 1.342684 | 5.16E-05 |
| GRP | 4.55E-07 | 1.288769 | 1.16259 | 1.428642 | 1.40E-06 |
| HSPA2 | 0.000488 | 1.308978 | 1.150265 | 1.489591 | 4.45E-05 |
| HSPB2 | 0.000163 | 1.400439 | 1.166647 | 1.681083 | 0.000302 |
| HSPB6 | 8.39E-08 | 1.148816 | 1.069491 | 1.234024 | 0.000145 |
| HSPB7 | 1.80E-06 | 1.206694 | 1.10799 | 1.314192 | 1.59E-05 |
| IL21R | 0.000272 | 0.313799 | 0.178729 | 0.550943 | 5.44E-05 |
| IL28B | 3.27E-06 | 1.222287 | 1.097019 | 1.36186 | 0.000274 |
| ISLR | 0.00021 | 1.336689 | 1.131944 | 1.578468 | 0.000624 |
| ITGB1 | 2.36E-05 | 2.036512 | 1.509533 | 2.747459 | 3.23E-06 |
| ITGB5 | 0.000571 | 1.850968 | 1.428285 | 2.398739 | 3.24E-06 |
| JAM3 | 0.000205 | 1.241142 | 1.099217 | 1.401392 | 0.000489 |
| KBTBD9 | 0.000275 | 1.645644 | 1.228054 | 2.205232 | 0.000851 |
| KCNIP3 | 0.000165 | 1.634098 | 1.21996 | 2.188823 | 0.00099 |
| KCNMB1 | 7.48E-06 | 1.148548 | 1.065881 | 1.237627 | 0.000279 |
| KCNS3 | 0.000505 | 1.467479 | 1.244188 | 1.730843 | 5.26E-06 |
| KIAA0367 | 0.000211 | 1.139361 | 1.057874 | 1.227125 | 0.000569 |
| KIAA1881 | 2.44E-06 | 1.168297 | 1.075483 | 1.269121 | 0.000231 |
| KIR2DL4 | 0.000605 | 0.62195 | 0.478916 | 0.807701 | 0.000369 |
| KLK11 | 8.35E-05 | 1.1565 | 1.070146 | 1.249822 | 0.00024 |
| KRT17 | 0.000153 | 1.142826 | 1.07428 | 1.215746 | 2.33E-05 |
| KRT7 | 0.000986 | 1.196373 | 1.106518 | 1.293525 | 6.77E-06 |
| LEPR | 0.000865 | 1.327459 | 1.14131 | 1.543969 | 0.000238 |
| LEPROT | 0.0008 | 1.618907 | 1.221658 | 2.14533 | 0.000797 |
| LETM1 | 0.000799 | 0.608223 | 0.465343 | 0.794972 | 0.000273 |
| LIMS2 | 0.000126 | 1.241025 | 1.097596 | 1.403197 | 0.000569 |
| LMO3 | 7.98E-07 | 1.262562 | 1.104044 | 1.443841 | 0.000659 |
| LMOD1 | 2.35E-08 | 1.19755 | 1.09559 | 1.308999 | 7.16E-05 |
| LOC401093 | 0.00018 | 1.215439 | 1.085566 | 1.360848 | 0.000714 |
| LOC642347 | 0.000555 | 4.30653 | 1.880707 | 9.861292 | 0.000552 |
| LOC649616 | 0.000469 | 4.577857 | 1.923678 | 10.89412 | 0.000584 |
| LOC650517 | 0.000137 | 1.365154 | 1.166913 | 1.597074 | 0.000101 |
| LOXL4 | 1.15E-05 | 1.389522 | 1.234119 | 1.564494 | 5.44E-08 |
| LPHN2 | 0.000535 | 1.337257 | 1.128496 | 1.584636 | 0.000791 |
| LPP | 6.50E-05 | 1.402196 | 1.182042 | 1.663353 | 0.000105 |
| LTBP3 | 0.000401 | 1.447695 | 1.199411 | 1.747374 | 0.000116 |
| MAMDC2 | 0.000597 | 1.170995 | 1.070254 | 1.281218 | 0.000583 |
| MAP1B | 0.000185 | 1.303734 | 1.130421 | 1.503618 | 0.000268 |
| MAPK4 | 0.000186 | 1.813558 | 1.32484 | 2.482558 | 0.000203 |
| MGLL | 0.000171 | 1.338273 | 1.129161 | 1.58611 | 0.000776 |
| MSRB3 | 5.33E-06 | 1.230955 | 1.110107 | 1.364959 | 8.11E-05 |
| MXRA7 | 4.42E-05 | 1.343723 | 1.150658 | 1.569183 | 0.000189 |
| MYADM | 1.25E-05 | 1.453789 | 1.215417 | 1.738911 | 4.22E-05 |
| MYH11 | 3.74E-08 | 1.177542 | 1.095719 | 1.265474 | 8.68E-06 |
| MYL9 | 1.84E-07 | 1.206854 | 1.110142 | 1.311992 | 1.03E-05 |
| MYLK | 3.30E-05 | 1.193006 | 1.097681 | 1.29661 | 3.27E-05 |
| NBEA | 0.000172 | 1.299282 | 1.129059 | 1.495169 | 0.000258 |
| NOV | 4.61E-07 | 1.475238 | 1.278902 | 1.701717 | 9.50E-08 |
| NUAK1 | 0.000394 | 1.454935 | 1.194659 | 1.771918 | 0.000193 |
| NUP88 | 0.00054 | 0.535446 | 0.385071 | 0.744544 | 0.000204 |
| PATL1 | 0.000148 | 0.630231 | 0.479942 | 0.827582 | 0.000895 |
| PDLIM3 | 0.000549 | 1.184602 | 1.081807 | 1.297164 | 0.000254 |
| PI4K2B | 0.000869 | 0.626707 | 0.505137 | 0.777533 | 2.17E-05 |
| PLCH1 | 5.06E-05 | 0.626638 | 0.507671 | 0.773484 | 1.35E-05 |
| PLN | 3.24E-05 | 1.306847 | 1.133545 | 1.506644 | 0.000227 |
| PNCK | 3.60E-05 | 1.133407 | 1.056533 | 1.215875 | 0.000475 |
| POLA2 | 0.0006 | 0.671698 | 0.533041 | 0.846423 | 0.000743 |
| PPP1R14A | 0.00026 | 1.261854 | 1.140238 | 1.396442 | 6.86E-06 |
| PPP1R3C | 0.000149 | 1.170107 | 1.073903 | 1.27493 | 0.000332 |
| PRKCDBP | 0.000862 | 1.455582 | 1.249267 | 1.695969 | 1.48E-06 |
| PSMB10 | 0.00056 | 0.671882 | 0.553311 | 0.815863 | 5.96E-05 |
| PTPN22 | 0.000585 | 0.563799 | 0.426062 | 0.746063 | 6.08E-05 |
| PTPRM | 4.01E-05 | 1.484328 | 1.244444 | 1.770453 | 1.13E-05 |
| QARS | 3.24E-05 | 0.543302 | 0.395302 | 0.746714 | 0.00017 |
| RAB23 | 0.000322 | 1.390986 | 1.186366 | 1.630898 | 4.80E-05 |
| RBPMS2 | 5.54E-07 | 1.19078 | 1.097582 | 1.291893 | 2.68E-05 |
| RTN4IP1 | 0.000849 | 0.57207 | 0.435352 | 0.751724 | 6.12E-05 |
| SAMD10 | 0.000713 | 0.495821 | 0.332857 | 0.738572 | 0.00056 |
| SCHIP1 | 0.00015 | 1.464429 | 1.225468 | 1.749985 | 2.71E-05 |
| SCLY | 8.30E-05 | 0.50872 | 0.374756 | 0.690572 | 1.46E-05 |
| SCRG1 | 2.95E-05 | 1.153096 | 1.08197 | 1.228899 | 1.16E-05 |
| SELM | 0.000881 | 1.313896 | 1.153137 | 1.497065 | 4.14E-05 |
| SEPSECS | 3.30E-07 | 0.400307 | 0.268947 | 0.595827 | 6.43E-06 |
| SFRP2 | 0.000837 | 1.158396 | 1.071874 | 1.251902 | 0.000205 |
| SFXN2 | 4.00E-05 | 0.554202 | 0.414575 | 0.740853 | 6.74E-05 |
| SGCE | 9.31E-05 | 1.303697 | 1.147675 | 1.480929 | 4.54E-05 |
| SLA2 | 0.00098 | 0.327723 | 0.195654 | 0.548939 | 2.25E-05 |
| SLC24A3 | 0.000253 | 1.387303 | 1.170139 | 1.64477 | 0.000164 |
| SLC27A2 | 0.00019 | 0.750817 | 0.660001 | 0.854129 | 1.32E-05 |
| SMARCAD1 | 0.000117 | 0.478695 | 0.335035 | 0.683956 | 5.20E-05 |
| SMYD1 | 0.000223 | 1.163182 | 1.065305 | 1.270052 | 0.00075 |
| SNORD43 | 0.000154 | 0.573452 | 0.437408 | 0.751809 | 5.71E-05 |
| SOD3 | 0.000531 | 1.240992 | 1.092641 | 1.409486 | 0.000888 |
| SORBS1 | 3.57E-07 | 1.236834 | 1.116079 | 1.370655 | 5.01E-05 |
| SOX15 | 0.000204 | 1.290951 | 1.118915 | 1.489437 | 0.000466 |
| SPARCL1 | 0.000638 | 1.196167 | 1.085889 | 1.317645 | 0.000284 |
| SPIRE1 | 0.000448 | 1.405454 | 1.205948 | 1.637965 | 1.32E-05 |
| SRRM1 | 1.32E-05 | 0.409449 | 0.277908 | 0.603252 | 6.30E-06 |
| STX17 | 6.11E-05 | 0.515707 | 0.351372 | 0.756901 | 0.000718 |
| STX18 | 0.000251 | 0.405046 | 0.252656 | 0.649351 | 0.000175 |
| SVIL | 0.000153 | 1.34144 | 1.163721 | 1.5463 | 5.10E-05 |
| SYNC1 | 7.91E-05 | 1.249406 | 1.117107 | 1.397374 | 9.65E-05 |
| SYNPO2 | 1.28E-06 | 1.231725 | 1.103708 | 1.37459 | 0.000197 |
| SYT17 | 0.000331 | 1.380063 | 1.151249 | 1.654354 | 0.000496 |
| TAGLN | 1.82E-05 | 1.311848 | 1.17141 | 1.469123 | 2.62E-06 |
| TCEAL2 | 6.42E-06 | 1.16671 | 1.083621 | 1.256169 | 4.30E-05 |
| TGFB1I1 | 5.36E-05 | 1.308933 | 1.137389 | 1.50635 | 0.000173 |
| TGFB2 | 0.000898 | 1.618692 | 1.25268 | 2.091648 | 0.000231 |
| TGFB3 | 0.00072 | 1.291641 | 1.119406 | 1.490377 | 0.000457 |
| THBS4 | 0.000229 | 1.122353 | 1.055158 | 1.193828 | 0.000248 |
| TICAM1 | 0.000402 | 0.669526 | 0.531359 | 0.843619 | 0.000669 |
| TMOD1 | 1.23E-06 | 1.237667 | 1.098663 | 1.394257 | 0.000452 |
| TPM1 | 0.000341 | 1.29048 | 1.14114 | 1.459365 | 4.82E-05 |
| TPM2 | 4.12E-05 | 1.310537 | 1.172009 | 1.465438 | 2.09E-06 |
| TRAF3 | 0.000296 | 0.579 | 0.438939 | 0.763754 | 0.00011 |
| TUBB6 | 0.000348 | 1.45088 | 1.226248 | 1.716662 | 1.45E-05 |
| VCL | 0.000344 | 1.715884 | 1.36972 | 2.149532 | 2.65E-06 |
| VSTM2L | 0.000459 | 1.475094 | 1.219558 | 1.784173 | 6.20E-05 |
| WNT7B | 4.74E-05 | 1.653481 | 1.307089 | 2.09167 | 2.76E-05 |
| ZNF101 | 5.47E-06 | 0.548636 | 0.394893 | 0.762236 | 0.000346 |
| ZNF232 | 7.06E-05 | 0.596118 | 0.45049 | 0.788822 | 0.000295 |
