# Supplementary figures and images for: Comprehensive Analysis of the Expression and Prognosis for ITGBs: Identification of ITGB5 as a Biomarker of Poor Prognosis and Correlated with Immune Infiltrates in Gastric Cancer
Source: Front Cell Dev Biol. 2022 Feb 9;9:816230. doi: 10.3389/fcell.2021.816230 (PMC8863963; doi:10.3389/fcell.2021.816230)

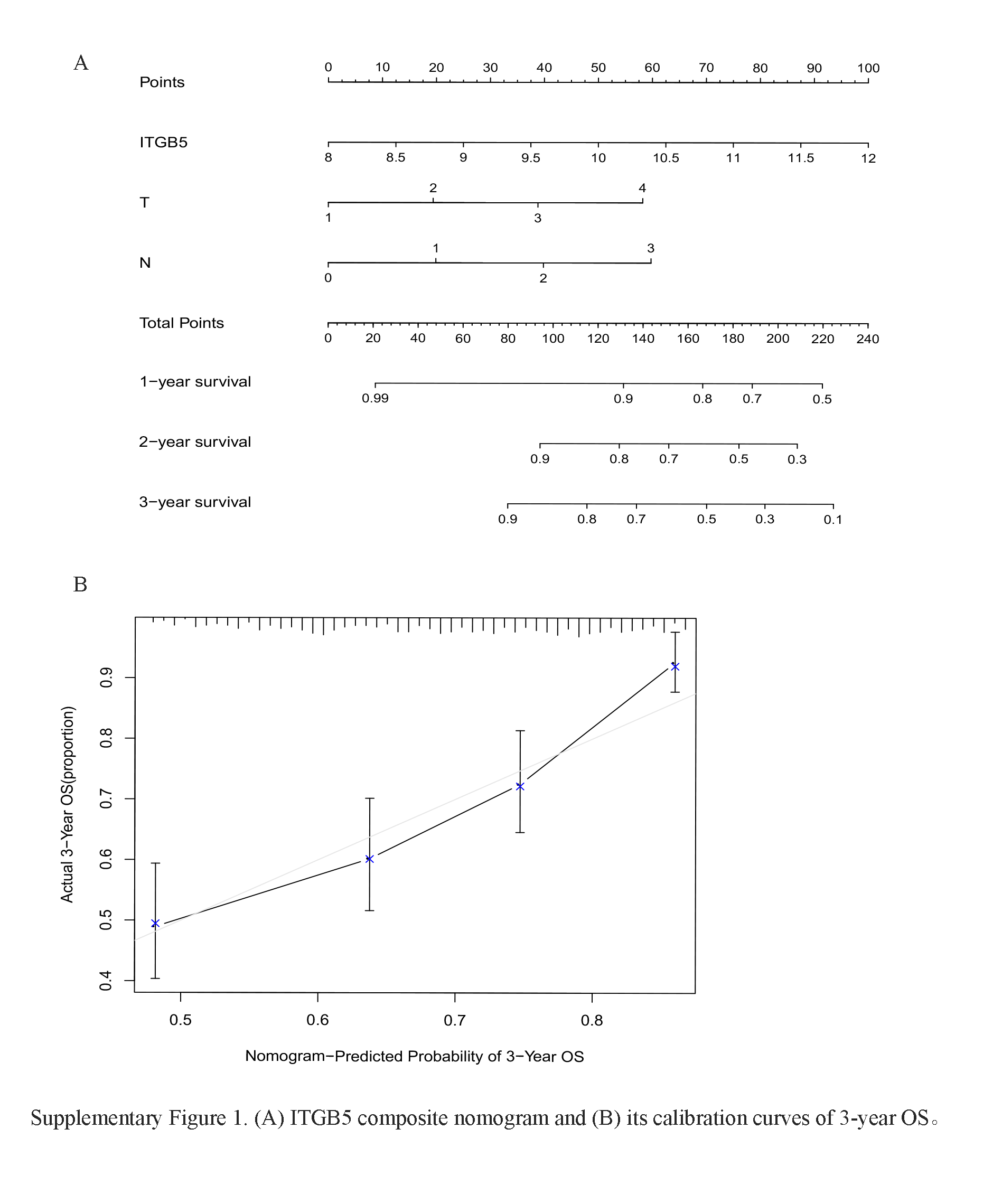

Supplement: Supplementary file 2 [file Image1.TIF]
