# Supplementary material for: Comprehensive Analysis of the Expression and Prognosis for ITGBs: Identification of ITGB5 as a Biomarker of Poor Prognosis and Correlated with Immune Infiltrates in Gastric Cancer
Source: Front Cell Dev Biol. 2022 Feb 9;9:816230. doi: 10.3389/fcell.2021.816230 (PMC8863963; doi:10.3389/fcell.2021.816230)
Supplement: Supplementary file 3 [file Table2.DOCX]

Supplementary Table 2 Differentially expressed and prognostic GC-related genes

| id | logFC | AveExpr | t | P.Value | adj.P.Val | B |
| --- | --- | --- | --- | --- | --- | --- |
| ITGB5 | 0.869815 | 10.4081 | 25.68691 | 4.43E-89 | 1.11E-84 | 188.5959 |
| TIMP2 | 0.898832 | 10.33328 | 11.05337 | 3.46E-25 | 4.34E-21 | 46.25181 |
| ANGPTL2 | 1.037703 | 10.00417 | 10.80183 | 3.04E-24 | 2.54E-20 | 44.14008 |
| RNF144 | 0.699078 | 8.592248 | 10.45769 | 5.66E-23 | 3.55E-19 | 41.29587 |
| SFRP4 | 1.344714 | 8.431044 | 10.14249 | 7.87E-22 | 3.29E-18 | 38.73862 |
| EFEMP2 | 0.817182 | 9.495348 | 9.835822 | 9.72E-21 | 3.49E-17 | 36.29652 |
| FNDC1 | 1.274839 | 8.807456 | 9.742765 | 2.07E-20 | 6.48E-17 | 35.56473 |
| PRKD1 | 0.649298 | 7.284188 | 9.71597 | 2.56E-20 | 7.15E-17 | 35.35484 |
| PDGFRL | 0.969554 | 8.016073 | 9.620486 | 5.52E-20 | 1.39E-16 | 34.60987 |
| MYADM | 0.653812 | 10.23485 | 9.595858 | 6.72E-20 | 1.53E-16 | 34.41848 |
| FAM65A | 0.583641 | 9.945949 | 9.436196 | 2.39E-19 | 4.62E-16 | 33.18543 |
| PMEPA1 | 0.870162 | 9.311452 | 9.328772 | 5.58E-19 | 9.26E-16 | 32.36339 |
| NUAK1 | 0.565653 | 9.373367 | 9.314653 | 6.24E-19 | 9.26E-16 | 32.25581 |
| PLSCR3 | 0.578601 | 10.97946 | 9.314023 | 6.27E-19 | 9.26E-16 | 32.25101 |
| C20orf82 | 0.874243 | 7.182498 | 9.233853 | 1.17E-18 | 1.47E-15 | 31.64221 |
| FAP | 0.909665 | 8.495365 | 9.220613 | 1.30E-18 | 1.56E-15 | 31.542 |
| HOXB2 | 0.824286 | 8.222818 | 9.209664 | 1.42E-18 | 1.62E-15 | 31.45921 |
| TSPAN9 | 0.619172 | 11.05278 | 9.184612 | 1.72E-18 | 1.74E-15 | 31.27002 |
| PRKCDBP | 0.753593 | 10.16366 | 9.178826 | 1.80E-18 | 1.74E-15 | 31.22637 |
| ANXA5 | 0.521077 | 11.68444 | 9.137667 | 2.48E-18 | 2.26E-15 | 30.91641 |
| C14orf78 | 0.977361 | 8.408377 | 9.135318 | 2.53E-18 | 2.26E-15 | 30.89875 |
| SPOCK1 | 0.978209 | 8.496976 | 9.127389 | 2.69E-18 | 2.31E-15 | 30.83916 |
| C20orf39 | 0.637262 | 7.067612 | 9.123728 | 2.76E-18 | 2.31E-15 | 30.81165 |
| THBS2 | 1.071463 | 10.97063 | 9.118103 | 2.89E-18 | 2.31E-15 | 30.7694 |
| MXRA7 | 0.639092 | 7.950807 | 9.1154 | 2.95E-18 | 2.31E-15 | 30.74912 |
| RAB31 | 0.71466 | 11.67277 | 9.107672 | 3.13E-18 | 2.38E-15 | 30.69112 |
| CTHRC1 | 0.993432 | 10.48468 | 9.093201 | 3.50E-18 | 2.58E-15 | 30.5826 |
| FER1L3 | 0.690373 | 10.27131 | 9.087521 | 3.66E-18 | 2.62E-15 | 30.54004 |
| SH3PXD2A | 0.59173 | 10.94738 | 8.992054 | 7.62E-18 | 5.28E-15 | 29.82737 |
| COL8A1 | 0.968883 | 9.155907 | 8.989433 | 7.78E-18 | 5.28E-15 | 29.80788 |
| COL12A1 | 0.728766 | 8.471187 | 8.981373 | 8.27E-18 | 5.46E-15 | 29.74795 |
| COL10A1 | 1.245544 | 8.287801 | 8.877517 | 1.83E-17 | 1.15E-14 | 28.9791 |
| GAS1 | 0.953067 | 8.239436 | 8.849254 | 2.27E-17 | 1.39E-14 | 28.77093 |
| SULF1 | 1.062289 | 11.00432 | 8.717398 | 6.13E-17 | 3.58E-14 | 27.8058 |
| GLT8D2 | 0.741118 | 8.524049 | 8.682719 | 7.95E-17 | 4.54E-14 | 27.55364 |
| PMP22 | 0.681727 | 10.02332 | 8.65795 | 9.57E-17 | 5.34E-14 | 27.37396 |
| LGALS1 | 0.683138 | 12.2874 | 8.604671 | 1.42E-16 | 7.77E-14 | 26.98869 |
| MFGE8 | 0.6333 | 12.01785 | 8.555525 | 2.05E-16 | 1.10E-13 | 26.63478 |
| CAV2 | 0.73011 | 8.310466 | 8.515842 | 2.75E-16 | 1.44E-13 | 26.35007 |
| CORIN | 0.553019 | 6.549461 | 8.489394 | 3.35E-16 | 1.68E-13 | 26.16082 |
| DKK3 | 0.653282 | 10.26493 | 8.425006 | 5.37E-16 | 2.59E-13 | 25.70187 |
| CDH11 | 0.773272 | 10.53889 | 8.422846 | 5.46E-16 | 2.59E-13 | 25.68651 |
| PAM | 0.533701 | 12.32437 | 8.418381 | 5.64E-16 | 2.62E-13 | 25.65479 |
| FRMD6 | 0.62193 | 7.876021 | 8.399335 | 6.49E-16 | 2.91E-13 | 25.51958 |
| PTRF | 0.663743 | 11.50052 | 8.32461 | 1.12E-15 | 4.84E-13 | 24.99123 |
| COL5A1 | 0.745147 | 10.90349 | 8.291029 | 1.43E-15 | 6.08E-13 | 24.75489 |
| BAG3 | 0.50014 | 10.10337 | 8.128972 | 4.59E-15 | 1.84E-12 | 23.62411 |
| PRRX1 | 0.749548 | 8.499303 | 8.128117 | 4.62E-15 | 1.84E-12 | 23.61818 |
| CCDC102A | 0.512851 | 8.597565 | 8.103925 | 5.49E-15 | 2.15E-12 | 23.45079 |
| TPST1 | 0.577354 | 8.318101 | 8.097276 | 5.76E-15 | 2.22E-12 | 23.40484 |
| ACTA2 | 0.752016 | 13.70304 | 8.08495 | 6.29E-15 | 2.39E-12 | 23.31974 |
| ACTN1 | 0.575423 | 12.13733 | 8.076759 | 6.67E-15 | 2.50E-12 | 23.26324 |
| CRABP2 | 0.926216 | 7.704812 | 7.994192 | 1.20E-14 | 4.06E-12 | 22.69609 |
| FAM127A | 0.61471 | 9.992869 | 7.992336 | 1.21E-14 | 4.06E-12 | 22.68339 |
| MSRB3 | 0.846897 | 8.018341 | 7.973901 | 1.38E-14 | 4.51E-12 | 22.55736 |
| CACNB3 | 0.521896 | 9.118256 | 7.947857 | 1.66E-14 | 5.34E-12 | 22.37969 |
| SGCE | 0.774138 | 9.641083 | 7.930001 | 1.88E-14 | 5.93E-12 | 22.25812 |
| BAPX1 | 0.992767 | 7.703859 | 7.929387 | 1.89E-14 | 5.93E-12 | 22.25394 |
| GJC2 | 0.63491 | 7.878203 | 7.922767 | 1.98E-14 | 6.07E-12 | 22.20893 |
| COL1A2 | 0.826522 | 12.37333 | 7.913 | 2.12E-14 | 6.42E-12 | 22.14257 |
| BGN | 0.645914 | 13.40628 | 7.827378 | 3.86E-14 | 1.10E-11 | 21.56339 |
| ANTXR1 | 0.609242 | 9.657596 | 7.781254 | 5.32E-14 | 1.47E-11 | 21.25335 |
| ITGA11 | 0.573703 | 7.312561 | 7.74226 | 6.97E-14 | 1.90E-11 | 20.99231 |
| RCN3 | 0.514649 | 8.156585 | 7.724919 | 7.86E-14 | 2.11E-11 | 20.87653 |
| C5orf13 | 0.624941 | 10.15544 | 7.723928 | 7.91E-14 | 2.11E-11 | 20.86992 |
| HOXA5 | 0.713428 | 10.04509 | 7.719836 | 8.14E-14 | 2.11E-11 | 20.84263 |
| IGFBP6 | 0.769361 | 9.093954 | 7.717674 | 8.26E-14 | 2.12E-11 | 20.82822 |
| C7orf10 | 0.539821 | 7.212714 | 7.708916 | 8.77E-14 | 2.20E-11 | 20.76987 |
| LUM | 0.87337 | 11.62181 | 7.697758 | 9.47E-14 | 2.35E-11 | 20.69559 |
| GFPT2 | 0.566824 | 7.168105 | 7.681352 | 1.06E-13 | 2.52E-11 | 20.58653 |
| NXN | 0.606016 | 8.440303 | 7.680983 | 1.06E-13 | 2.52E-11 | 20.58408 |
| TIMP3 | 0.786852 | 9.187747 | 7.634625 | 1.46E-13 | 3.36E-11 | 20.27688 |
| EPDR1 | 0.837057 | 9.698298 | 7.617697 | 1.64E-13 | 3.68E-11 | 20.16505 |
| PLSCR4 | 0.62046 | 8.852676 | 7.611517 | 1.71E-13 | 3.80E-11 | 20.12427 |
| PHLDB1 | 0.572527 | 9.778918 | 7.586699 | 2.03E-13 | 4.42E-11 | 19.96077 |
| EPAS1 | 0.523637 | 10.94543 | 7.585427 | 2.04E-13 | 4.42E-11 | 19.9524 |
| VCAN | 0.68647 | 10.24764 | 7.57097 | 2.26E-13 | 4.76E-11 | 19.85735 |
| IGFBP7 | 0.743569 | 12.44931 | 7.569581 | 2.28E-13 | 4.76E-11 | 19.84823 |
| MOXD1 | 0.711779 | 8.155867 | 7.537704 | 2.83E-13 | 5.86E-11 | 19.63917 |
| SPARC | 0.737322 | 11.37007 | 7.535007 | 2.88E-13 | 5.91E-11 | 19.62151 |
| DCN | 0.802667 | 11.73057 | 7.530576 | 2.97E-13 | 6.00E-11 | 19.59251 |
| AXL | 0.509652 | 9.033298 | 7.501645 | 3.61E-13 | 7.13E-11 | 19.4035 |
| C13orf33 | 0.528698 | 6.803829 | 7.493477 | 3.81E-13 | 7.47E-11 | 19.35024 |
| THBS1 | 0.800967 | 10.50979 | 7.486908 | 3.98E-13 | 7.75E-11 | 19.30743 |
| A4GALT | 0.522182 | 7.702152 | 7.482775 | 4.10E-13 | 7.91E-11 | 19.28052 |
| TUBB6 | 0.517266 | 8.069341 | 7.465409 | 4.60E-13 | 8.76E-11 | 19.16755 |
| C1QTNF5 | 0.658865 | 9.8562 | 7.408566 | 6.74E-13 | 1.27E-10 | 18.79918 |
| GEM | 0.577294 | 8.006283 | 7.396351 | 7.32E-13 | 1.34E-10 | 18.7203 |
| CAP2 | 0.75361 | 9.082341 | 7.384907 | 7.90E-13 | 1.42E-10 | 18.6465 |
| RAB3IL1 | 0.541659 | 9.010928 | 7.361598 | 9.23E-13 | 1.63E-10 | 18.49644 |
| HES4 | 0.593807 | 10.45001 | 7.348559 | 1.01E-12 | 1.73E-10 | 18.41266 |
| FBXO32 | 0.611176 | 8.928344 | 7.279642 | 1.59E-12 | 2.66E-10 | 17.97176 |
| CALD1 | 0.744105 | 10.88167 | 7.264527 | 1.76E-12 | 2.90E-10 | 17.87549 |
| DCBLD2 | 0.643794 | 9.091218 | 7.249872 | 1.93E-12 | 3.13E-10 | 17.7823 |
| DPYSL3 | 0.847243 | 11.33854 | 7.246304 | 1.98E-12 | 3.18E-10 | 17.75963 |
| MPDZ | 0.570618 | 7.618523 | 7.24308 | 2.02E-12 | 3.23E-10 | 17.73916 |
| TSHZ3 | 0.580467 | 8.408414 | 7.231356 | 2.18E-12 | 3.45E-10 | 17.66477 |
| FBLN2 | 0.708902 | 8.731494 | 7.164761 | 3.38E-12 | 5.14E-10 | 17.24399 |
| OLFML1 | 0.507698 | 7.690094 | 7.163884 | 3.40E-12 | 5.14E-10 | 17.23847 |
| LARP6 | 0.571023 | 7.860407 | 7.161346 | 3.45E-12 | 5.19E-10 | 17.2225 |
| RGS4 | 0.665352 | 7.803716 | 7.151057 | 3.69E-12 | 5.45E-10 | 17.15778 |
| NOX4 | 0.600327 | 8.424377 | 7.147991 | 3.77E-12 | 5.48E-10 | 17.13851 |
| ZBTB20 | 0.526776 | 9.005429 | 7.147485 | 3.78E-12 | 5.48E-10 | 17.13534 |
| TAGLN | 0.817374 | 11.90016 | 7.13081 | 4.21E-12 | 6.04E-10 | 17.03065 |
| CD248 | 0.638696 | 9.565939 | 7.127339 | 4.31E-12 | 6.15E-10 | 17.00888 |
| PALM | 0.540129 | 7.561085 | 7.122394 | 4.45E-12 | 6.31E-10 | 16.97789 |
| SFRP2 | 1.179183 | 9.604504 | 7.121287 | 4.48E-12 | 6.32E-10 | 16.97095 |
| OMD | 0.706139 | 7.227916 | 7.118436 | 4.57E-12 | 6.40E-10 | 16.95309 |
| FSCN1 | 0.620461 | 11.08351 | 7.099125 | 5.18E-12 | 7.16E-10 | 16.83227 |
| LRRC32 | 0.604992 | 9.6456 | 7.098713 | 5.19E-12 | 7.16E-10 | 16.82969 |
| ARMCX1 | 0.525647 | 7.676234 | 7.08204 | 5.78E-12 | 7.93E-10 | 16.72558 |
| PDPN | 0.552788 | 8.591086 | 7.070351 | 6.23E-12 | 8.41E-10 | 16.65271 |
| NCAPG | -0.59974 | 9.269341 | -7.0673 | 6.36E-12 | 8.53E-10 | 16.63372 |
| NDN | 0.773561 | 9.898555 | 7.061202 | 6.61E-12 | 8.78E-10 | 16.59574 |
| RAB34 | 0.637114 | 8.751156 | 7.055077 | 6.88E-12 | 8.99E-10 | 16.55763 |
| TGFB1I1 | 0.534446 | 7.722696 | 7.046737 | 7.26E-12 | 9.35E-10 | 16.50578 |
| FAM150A | 0.706179 | 7.502521 | 7.031242 | 8.02E-12 | 1.02E-09 | 16.40959 |
| PCOLCE | 0.685763 | 8.854238 | 7.027478 | 8.22E-12 | 1.04E-09 | 16.38625 |
| RARRES2 | 0.666661 | 10.22609 | 7.00205 | 9.68E-12 | 1.20E-09 | 16.22881 |
| CYBRD1 | 0.791257 | 10.39924 | 6.999975 | 9.81E-12 | 1.21E-09 | 16.21598 |
| MYH10 | 0.703414 | 10.41271 | 6.998117 | 9.92E-12 | 1.22E-09 | 16.2045 |
| GALNAC4S-6ST | 0.584333 | 9.524589 | 6.975611 | 1.15E-11 | 1.37E-09 | 16.06559 |
| MXRA5 | 0.614427 | 11.97023 | 6.960936 | 1.26E-11 | 1.48E-09 | 15.97521 |
| TGFB3 | 0.594206 | 8.624347 | 6.924106 | 1.59E-11 | 1.79E-09 | 15.74905 |
| LOXL4 | 0.664141 | 7.681561 | 6.912066 | 1.72E-11 | 1.90E-09 | 15.67532 |
| PALLD | 0.594484 | 11.57644 | 6.894559 | 1.92E-11 | 2.10E-09 | 15.5683 |
| C14orf132 | 0.726807 | 8.252342 | 6.873636 | 2.19E-11 | 2.36E-09 | 15.44068 |
| ECM2 | 0.535901 | 8.019561 | 6.867019 | 2.29E-11 | 2.39E-09 | 15.40039 |
| KIF15 | -0.53823 | 8.413711 | -6.86697 | 2.29E-11 | 2.39E-09 | 15.40011 |
| CUEDC1 | 0.545792 | 10.17896 | 6.855647 | 2.46E-11 | 2.54E-09 | 15.3312 |
| SLIT2 | 0.696412 | 8.081871 | 6.847638 | 2.58E-11 | 2.65E-09 | 15.28253 |
| TPM2 | 0.74284 | 11.88096 | 6.819673 | 3.08E-11 | 3.08E-09 | 15.11296 |
| FLJ35258 | 0.682906 | 7.995519 | 6.808902 | 3.30E-11 | 3.27E-09 | 15.0478 |
| PDLIM3 | 0.910219 | 9.81985 | 6.794963 | 3.60E-11 | 3.55E-09 | 14.96359 |
| TPM1 | 0.627904 | 10.64882 | 6.790252 | 3.70E-11 | 3.65E-09 | 14.93517 |
| KANK2 | 0.610626 | 7.954471 | 6.779725 | 3.96E-11 | 3.86E-09 | 14.8717 |
| SNCA | 0.55338 | 7.076864 | 6.763332 | 4.38E-11 | 4.22E-09 | 14.77302 |
| FERMT2 | 0.640029 | 10.81253 | 6.755658 | 4.60E-11 | 4.37E-09 | 14.72689 |
| LAMA5 | 0.525857 | 11.89092 | 6.745071 | 4.91E-11 | 4.60E-09 | 14.66333 |
| LOX | 0.554321 | 7.428134 | 6.730774 | 5.37E-11 | 4.94E-09 | 14.57761 |
| RASL12 | 0.501763 | 8.029722 | 6.727686 | 5.47E-11 | 5.02E-09 | 14.55912 |
| CTSK | 0.670898 | 10.38839 | 6.687406 | 7.03E-11 | 6.37E-09 | 14.3185 |
| FBLN1 | 0.838594 | 10.16161 | 6.683097 | 7.22E-11 | 6.52E-09 | 14.29284 |
| MFAP4 | 0.935827 | 10.85531 | 6.662831 | 8.18E-11 | 7.23E-09 | 14.17228 |
| LAYN | 0.512103 | 7.417734 | 6.662327 | 8.21E-11 | 7.23E-09 | 14.16929 |
| SOX18 | 0.566181 | 10.08034 | 6.649487 | 8.88E-11 | 7.72E-09 | 14.09307 |
| COMP | 0.78062 | 7.357682 | 6.637184 | 9.58E-11 | 8.21E-09 | 14.02014 |
| COL5A2 | 0.550084 | 11.66854 | 6.63481 | 9.72E-11 | 8.30E-09 | 14.00609 |
| MMP11 | 0.992531 | 9.778804 | 6.609143 | 1.14E-10 | 9.59E-09 | 13.85435 |
| PBX3 | 0.546341 | 10.06321 | 6.604708 | 1.17E-10 | 9.80E-09 | 13.82818 |
| FXYD6 | 0.592322 | 7.767064 | 6.604494 | 1.17E-10 | 9.80E-09 | 13.82692 |
| ITGA5 | 0.536486 | 9.367861 | 6.599195 | 1.21E-10 | 1.01E-08 | 13.79567 |
| HOPX | 0.686679 | 8.677856 | 6.594631 | 1.24E-10 | 1.03E-08 | 13.76877 |
| CLIP3 | 0.734433 | 9.552463 | 6.590035 | 1.28E-10 | 1.06E-08 | 13.7417 |
| COL1A1 | 0.803566 | 12.45174 | 6.579357 | 1.37E-10 | 1.12E-08 | 13.67887 |
| TNC | 0.692054 | 8.995873 | 6.562982 | 1.51E-10 | 1.23E-08 | 13.58267 |
| KIAA0644 | 0.522585 | 7.786605 | 6.558713 | 1.55E-10 | 1.26E-08 | 13.55762 |
| SCRN1 | 0.585102 | 9.648605 | 6.556722 | 1.57E-10 | 1.27E-08 | 13.54594 |
| CAV1 | 0.6922 | 9.93326 | 6.545823 | 1.68E-10 | 1.34E-08 | 13.48207 |
| CYR61 | 0.665236 | 9.700682 | 6.531551 | 1.83E-10 | 1.44E-08 | 13.39857 |
| SPON1 | 0.891645 | 9.947964 | 6.52848 | 1.86E-10 | 1.46E-08 | 13.38062 |
| ODZ3 | 0.593824 | 7.442357 | 6.513471 | 2.04E-10 | 1.56E-08 | 13.293 |
| DUSP1 | 0.573267 | 11.90792 | 6.494049 | 2.30E-10 | 1.73E-08 | 13.17985 |
| VASN | 0.5187 | 9.408892 | 6.490058 | 2.35E-10 | 1.76E-08 | 13.15664 |
| COL15A1 | 0.602561 | 10.29435 | 6.488316 | 2.38E-10 | 1.77E-08 | 13.1465 |
| NNMT | 0.513386 | 9.163794 | 6.48746 | 2.39E-10 | 1.77E-08 | 13.14153 |
| ARHGEF10 | 0.543392 | 9.294264 | 6.47111 | 2.64E-10 | 1.94E-08 | 13.04658 |
| DACT3 | 0.743229 | 8.994069 | 6.466199 | 2.72E-10 | 1.99E-08 | 13.01809 |
| RBPMS2 | 0.89616 | 9.469343 | 6.461674 | 2.79E-10 | 2.04E-08 | 12.99187 |
| HTRA1 | 0.570933 | 11.03008 | 6.457091 | 2.87E-10 | 2.09E-08 | 12.96532 |
| ROR2 | 0.542902 | 7.786558 | 6.450306 | 2.99E-10 | 2.15E-08 | 12.92604 |
| KAL1 | 0.525451 | 7.563755 | 6.448591 | 3.02E-10 | 2.16E-08 | 12.91612 |
| VIM | 0.511634 | 12.19919 | 6.448084 | 3.03E-10 | 2.16E-08 | 12.91319 |
| GNG12 | 0.51639 | 8.200487 | 6.438489 | 3.21E-10 | 2.27E-08 | 12.85772 |
| DFNA5 | 0.569371 | 8.756328 | 6.432219 | 3.33E-10 | 2.34E-08 | 12.82151 |
| RGMA | 0.939541 | 9.398617 | 6.422704 | 3.53E-10 | 2.46E-08 | 12.76661 |
| GRP | 0.657628 | 6.709758 | 6.418933 | 3.61E-10 | 2.51E-08 | 12.74488 |
| SERPINE1 | 0.588086 | 7.892222 | 6.405756 | 3.91E-10 | 2.69E-08 | 12.669 |
| TCEAL3 | 0.568763 | 9.956156 | 6.404508 | 3.94E-10 | 2.71E-08 | 12.66182 |
| GREM1 | 0.878068 | 10.04975 | 6.39811 | 4.09E-10 | 2.80E-08 | 12.62503 |
| FSTL1 | 0.552425 | 9.971188 | 6.393599 | 4.20E-10 | 2.86E-08 | 12.59911 |
| MFAP5 | 0.819336 | 9.180054 | 6.392352 | 4.23E-10 | 2.87E-08 | 12.59195 |
| CNTNAP1 | 0.51206 | 7.767229 | 6.380465 | 4.54E-10 | 3.06E-08 | 12.52373 |
| SPARCL1 | 0.840138 | 11.7327 | 6.378601 | 4.59E-10 | 3.08E-08 | 12.51305 |
| EFEMP1 | 0.718554 | 8.709677 | 6.369105 | 4.86E-10 | 3.23E-08 | 12.45864 |
| ARMCX2 | 0.546241 | 8.850014 | 6.339277 | 5.81E-10 | 3.79E-08 | 12.28817 |
| CTGF | 0.542807 | 12.15268 | 6.329392 | 6.16E-10 | 3.95E-08 | 12.23182 |
| PNMA2 | 0.610296 | 7.721199 | 6.318666 | 6.56E-10 | 4.19E-08 | 12.17076 |
| SYT11 | 0.582616 | 10.07259 | 6.311267 | 6.86E-10 | 4.36E-08 | 12.12869 |
| PRICKLE2 | 0.533113 | 8.492821 | 6.307165 | 7.02E-10 | 4.42E-08 | 12.10538 |
| MAFB | 0.535009 | 9.599485 | 6.26789 | 8.85E-10 | 5.42E-08 | 11.88288 |
| C1R | 0.619211 | 8.247332 | 6.238618 | 1.05E-09 | 6.24E-08 | 11.71779 |
| MGP | 0.938324 | 11.65144 | 6.223151 | 1.15E-09 | 6.69E-08 | 11.63082 |
| LOC387763 | 0.578465 | 9.419012 | 6.215331 | 1.21E-09 | 6.93E-08 | 11.58692 |
| MN1 | 0.537952 | 7.80651 | 6.196478 | 1.35E-09 | 7.62E-08 | 11.48127 |
| EFHD1 | 0.548026 | 8.199408 | 6.195195 | 1.36E-09 | 7.65E-08 | 11.47409 |
| STON1 | 0.545269 | 7.173776 | 6.195103 | 1.36E-09 | 7.65E-08 | 11.47357 |
| SVEP1 | 0.729109 | 8.76776 | 6.186793 | 1.42E-09 | 7.94E-08 | 11.42709 |
| COL3A1 | 0.556433 | 12.92861 | 6.175929 | 1.52E-09 | 8.42E-08 | 11.36641 |
| MRGPRF | 0.678019 | 7.673754 | 6.137064 | 1.90E-09 | 1.02E-07 | 11.15005 |
| CDH2 | 0.559115 | 8.045375 | 6.134669 | 1.93E-09 | 1.03E-07 | 11.13676 |
| ZAK | 0.574092 | 9.524413 | 6.129071 | 1.99E-09 | 1.06E-07 | 11.1057 |
| BNC2 | 0.50779 | 7.609766 | 6.117596 | 2.13E-09 | 1.11E-07 | 11.04211 |
| PTGIS | 0.673173 | 7.598746 | 6.110282 | 2.22E-09 | 1.16E-07 | 11.00163 |
| PDGFC | 0.500219 | 8.19015 | 6.1012 | 2.34E-09 | 1.21E-07 | 10.95142 |
| CST6 | 0.559566 | 6.52997 | 6.091595 | 2.47E-09 | 1.25E-07 | 10.89839 |
| JAM3 | 0.601427 | 10.01881 | 6.086247 | 2.55E-09 | 1.28E-07 | 10.86889 |
| KIAA1199 | 0.858175 | 9.446949 | 6.076369 | 2.70E-09 | 1.35E-07 | 10.81446 |
| CPXM2 | 0.76489 | 8.683822 | 6.063621 | 2.90E-09 | 1.44E-07 | 10.74433 |
| COL6A3 | 0.553535 | 12.09821 | 6.047922 | 3.17E-09 | 1.56E-07 | 10.65814 |
| C20orf103 | 0.578042 | 7.305737 | 6.046099 | 3.21E-09 | 1.58E-07 | 10.64814 |
| CACNA1H | 0.632303 | 8.065985 | 6.029644 | 3.52E-09 | 1.72E-07 | 10.55802 |
| RAB32 | 0.525724 | 9.655484 | 6.027478 | 3.57E-09 | 1.74E-07 | 10.54617 |
| EML1 | 0.505567 | 7.848853 | 6.013667 | 3.86E-09 | 1.87E-07 | 10.47072 |
| THBS4 | 1.203099 | 9.140352 | 6.013374 | 3.87E-09 | 1.87E-07 | 10.46912 |
| CBX6 | 0.560615 | 9.369545 | 5.995998 | 4.27E-09 | 2.02E-07 | 10.3744 |
| PROS1 | 0.569816 | 10.13639 | 5.992717 | 4.35E-09 | 2.04E-07 | 10.35654 |
| HEYL | 0.559974 | 9.63337 | 5.972857 | 4.87E-09 | 2.25E-07 | 10.24861 |
| PPP1R3C | 0.787144 | 8.282645 | 5.970844 | 4.92E-09 | 2.26E-07 | 10.23769 |
| CRYAB | 0.752625 | 8.389546 | 5.963113 | 5.14E-09 | 2.35E-07 | 10.19576 |
| SPP1 | 0.988562 | 9.590526 | 5.954271 | 5.41E-09 | 2.45E-07 | 10.14787 |
| CRIP2 | 0.545774 | 9.693947 | 5.948689 | 5.58E-09 | 2.52E-07 | 10.11767 |
| PODN | 0.727954 | 9.441643 | 5.938739 | 5.90E-09 | 2.65E-07 | 10.06389 |
| S100A4 | 0.610253 | 11.57986 | 5.922002 | 6.48E-09 | 2.89E-07 | 9.973612 |
| CRISPLD2 | 0.560517 | 10.35448 | 5.90619 | 7.09E-09 | 3.11E-07 | 9.888518 |
| ADAMTSL2 | 0.509682 | 8.345055 | 5.90099 | 7.30E-09 | 3.18E-07 | 9.860574 |
| DIO2 | 0.557827 | 8.585434 | 5.896339 | 7.49E-09 | 3.24E-07 | 9.835596 |
| COLEC12 | 0.518908 | 7.524283 | 5.895181 | 7.54E-09 | 3.25E-07 | 9.829383 |
| PLAT | 0.556154 | 9.16577 | 5.853234 | 9.53E-09 | 3.93E-07 | 9.604927 |
| HSPB8 | 0.692617 | 7.709683 | 5.835802 | 1.05E-08 | 4.29E-07 | 9.512048 |
| MMP7 | 1.236074 | 11.00655 | 5.834249 | 1.06E-08 | 4.30E-07 | 9.503787 |
| KLK7 | 0.703245 | 7.392452 | 5.832434 | 1.07E-08 | 4.34E-07 | 9.494135 |
| AEBP1 | 0.624058 | 10.24717 | 5.755675 | 1.64E-08 | 6.32E-07 | 9.088177 |
| C10orf56 | 0.540849 | 8.953394 | 5.744476 | 1.74E-08 | 6.68E-07 | 9.029333 |
| GUCY1A3 | 0.639306 | 9.69892 | 5.736241 | 1.82E-08 | 6.94E-07 | 8.986127 |
| CLEC11A | 0.522944 | 7.80052 | 5.715341 | 2.04E-08 | 7.67E-07 | 8.876709 |
| SERPINF1 | 0.574211 | 8.687934 | 5.709618 | 2.11E-08 | 7.89E-07 | 8.846806 |
| ZSCAN18 | 0.638216 | 9.365411 | 5.692695 | 2.31E-08 | 8.52E-07 | 8.758533 |
| PRRX2 | 0.562179 | 8.315335 | 5.689155 | 2.35E-08 | 8.65E-07 | 8.7401 |
| MYL9 | 0.757428 | 8.921724 | 5.654802 | 2.84E-08 | 1.02E-06 | 8.561697 |
| OSR1 | 0.524499 | 7.527181 | 5.64166 | 3.05E-08 | 1.09E-06 | 8.493697 |
| MKX | 0.535818 | 7.401449 | 5.579819 | 4.25E-08 | 1.46E-06 | 8.175532 |
| SFTPG | 0.694106 | 7.891595 | 5.561941 | 4.68E-08 | 1.58E-06 | 8.084119 |
| LOC493869 | 0.502005 | 8.765755 | 5.527255 | 5.63E-08 | 1.87E-06 | 7.907483 |
| KRT7 | 0.918728 | 8.93029 | 5.518529 | 5.90E-08 | 1.94E-06 | 7.863194 |
| FREQ | 0.541467 | 7.974367 | 5.514007 | 6.04E-08 | 1.98E-06 | 7.840272 |
| MYH11 | 1.015524 | 11.49086 | 5.477737 | 7.32E-08 | 2.32E-06 | 7.656972 |
| CNN1 | 1.050702 | 8.998375 | 5.476279 | 7.38E-08 | 2.33E-06 | 7.649625 |
| NR2F1 | 0.589457 | 8.466723 | 5.472481 | 7.53E-08 | 2.36E-06 | 7.630496 |
| MSLN | 1.115871 | 10.35211 | 5.466235 | 7.78E-08 | 2.42E-06 | 7.599066 |
| IGFBP3 | 0.526558 | 10.76607 | 5.45405 | 8.29E-08 | 2.57E-06 | 7.537834 |
| LOC401093 | 0.515867 | 6.968047 | 5.452097 | 8.38E-08 | 2.59E-06 | 7.528033 |
| RHOD | 0.588332 | 8.723747 | 5.431891 | 9.32E-08 | 2.85E-06 | 7.426789 |
| KLK6 | 0.887423 | 7.788198 | 5.393354 | 1.14E-07 | 3.42E-06 | 7.234602 |
| CKMT1B | -0.52601 | 8.562594 | -5.36407 | 1.33E-07 | 3.93E-06 | 7.089373 |
| CHRDL2 | 0.625828 | 7.452105 | 5.362895 | 1.34E-07 | 3.95E-06 | 7.083545 |
| SYNC1 | 0.534168 | 7.779358 | 5.344364 | 1.47E-07 | 4.29E-06 | 6.99201 |
| TUBB3 | 0.533225 | 8.982587 | 5.320657 | 1.66E-07 | 4.77E-06 | 6.875306 |
| EMILIN1 | 0.545089 | 8.362485 | 5.314795 | 1.71E-07 | 4.89E-06 | 6.846523 |
| LHFP | 0.508584 | 8.868845 | 5.291049 | 1.94E-07 | 5.43E-06 | 6.730196 |
| COL11A1 | 0.585949 | 7.277833 | 5.288289 | 1.96E-07 | 5.49E-06 | 6.716708 |
| SHISA2 | 0.541823 | 7.965838 | 5.26962 | 2.16E-07 | 6.01E-06 | 6.625617 |
| GPNMB | 0.565191 | 10.06553 | 5.251807 | 2.37E-07 | 6.47E-06 | 6.538963 |
| CASP1 | -0.55508 | 9.830463 | -5.25174 | 2.37E-07 | 6.47E-06 | 6.538623 |
| C1S | 0.542543 | 10.06648 | 5.245058 | 2.45E-07 | 6.65E-06 | 6.5062 |
| GJA1 | 0.529367 | 9.386479 | 5.236402 | 2.56E-07 | 6.89E-06 | 6.464234 |
| APOD | 0.998977 | 9.960353 | 5.235751 | 2.57E-07 | 6.89E-06 | 6.461082 |
| COL16A1 | 0.516699 | 9.499036 | 5.235697 | 2.57E-07 | 6.89E-06 | 6.460818 |
| PPP1R14A | 0.582049 | 9.081383 | 5.21497 | 2.85E-07 | 7.50E-06 | 6.360588 |
| RNF150 | 0.569986 | 8.246935 | 5.162082 | 3.73E-07 | 9.42E-06 | 6.106423 |
| C15orf52 | 0.551298 | 9.691618 | 5.16198 | 3.73E-07 | 9.42E-06 | 6.105934 |
| SRPX | 0.644592 | 8.34913 | 5.152846 | 3.91E-07 | 9.78E-06 | 6.062271 |
| RERG | 0.548667 | 7.646861 | 5.130276 | 4.37E-07 | 1.08E-05 | 5.954674 |
| SMOC2 | 0.708547 | 9.10841 | 5.098049 | 5.14E-07 | 1.24E-05 | 5.801758 |
| KCNMB1 | 0.795873 | 9.41348 | 5.088375 | 5.39E-07 | 1.29E-05 | 5.756021 |
| CLDN1 | 0.638069 | 10.66877 | 5.025727 | 7.35E-07 | 1.69E-05 | 5.461704 |
| ACTG2 | 0.813621 | 12.52516 | 4.900545 | 1.35E-06 | 2.87E-05 | 4.883319 |
| COL6A2 | 0.512248 | 8.737822 | 4.886058 | 1.45E-06 | 3.05E-05 | 4.817224 |
| PNCK | 0.783364 | 7.981901 | 4.84102 | 1.80E-06 | 3.65E-05 | 4.612862 |
| CA12 | 0.570799 | 8.187007 | 4.825794 | 1.94E-06 | 3.89E-05 | 4.544156 |
| FOXC1 | 0.559147 | 10.83059 | 4.740659 | 2.90E-06 | 5.49E-05 | 4.163576 |
| HSPB7 | 0.609365 | 7.36134 | 4.739129 | 2.92E-06 | 5.53E-05 | 4.156789 |
| MYB | -0.53031 | 10.42941 | -4.71852 | 3.21E-06 | 6.01E-05 | 4.065624 |
| SERPINE2 | 0.5234 | 11.61935 | 4.717675 | 3.22E-06 | 6.03E-05 | 4.061871 |
| CAMK2N1 | 0.533026 | 11.77398 | 4.708602 | 3.36E-06 | 6.27E-05 | 4.021848 |
| RARRES1 | 0.576046 | 9.006381 | 4.67837 | 3.87E-06 | 7.04E-05 | 3.888984 |
| KRT17 | 0.936799 | 9.29156 | 4.650034 | 4.41E-06 | 7.84E-05 | 3.765153 |
| CST1 | 0.858663 | 10.05207 | 4.648572 | 4.44E-06 | 7.88E-05 | 3.75878 |
| MYLK | 0.634851 | 8.741621 | 4.63302 | 4.77E-06 | 8.31E-05 | 3.691126 |
| OLR1 | 0.510024 | 8.619482 | 4.563662 | 6.56E-06 | 0.000108 | 3.391898 |
| CASQ2 | 0.501165 | 6.911751 | 4.504104 | 8.58E-06 | 0.000136 | 3.138217 |
| POSTN | 0.512482 | 8.82167 | 4.482331 | 9.46E-06 | 0.000147 | 3.046231 |
| SCRG1 | 0.823888 | 8.228392 | 4.476552 | 9.71E-06 | 0.000151 | 3.021884 |
| LMOD1 | 0.529476 | 7.293408 | 4.401874 | 1.35E-05 | 0.000199 | 2.70985 |
| PI16 | 0.547131 | 7.384471 | 4.394249 | 1.40E-05 | 0.000204 | 2.678257 |
| FLJ10916 | 0.533611 | 8.616775 | 4.391234 | 1.42E-05 | 0.000206 | 2.665781 |
| SFRP1 | 0.662189 | 7.607604 | 4.360888 | 1.62E-05 | 0.000232 | 2.54063 |
| CTSG | 0.569275 | 7.399572 | 4.33935 | 1.78E-05 | 0.000251 | 2.452283 |
| MUC20 | 0.592651 | 8.759572 | 4.287108 | 2.23E-05 | 0.000303 | 2.23966 |
| FLNC | 0.611475 | 9.93092 | 4.284518 | 2.26E-05 | 0.000305 | 2.22918 |
| GPR128 | -0.63134 | 8.43454 | -4.25214 | 2.60E-05 | 0.000343 | 2.098671 |
| GZMB | -0.53042 | 9.01759 | -4.19446 | 3.32E-05 | 0.000418 | 1.868405 |
| SMYD1 | 0.501302 | 6.353475 | 4.184611 | 3.46E-05 | 0.000433 | 1.829376 |
| TCEAL2 | 0.648831 | 7.787228 | 4.121487 | 4.51E-05 | 0.000537 | 1.581248 |
| TACSTD2 | 0.572086 | 11.99414 | 4.090525 | 5.13E-05 | 0.000597 | 1.460813 |
| FCER1A | 0.502288 | 7.341564 | 4.044879 | 6.20E-05 | 0.000701 | 1.284798 |
| DKK1 | 0.635559 | 7.345904 | 4.002996 | 7.36E-05 | 0.000808 | 1.124898 |
| NKD2 | 0.53124 | 7.946536 | 3.981199 | 8.04E-05 | 0.000871 | 1.042294 |
| SCNN1A | 0.641169 | 10.99059 | 3.929358 | 9.91E-05 | 0.001033 | 0.847513 |
| PGM5 | 0.615732 | 8.537144 | 3.922441 | 0.000102 | 0.001058 | 0.821706 |
| MAMDC2 | 0.504804 | 7.558193 | 3.914758 | 0.000105 | 0.001088 | 0.793086 |
| KIAA1881 | 0.502201 | 7.23152 | 3.891312 | 0.000115 | 0.001177 | 0.706075 |
| ATP4A | -0.51733 | 6.451396 | -3.8468 | 0.000138 | 0.001359 | 0.542213 |
| PON3 | 0.541292 | 7.921514 | 3.808725 | 0.00016 | 0.001535 | 0.403469 |
| HSPB6 | 0.584086 | 7.97114 | 3.706826 | 0.000237 | 0.00212 | 0.038467 |
| C6orf105 | -0.50486 | 7.894693 | -3.66464 | 0.000278 | 0.002412 | -0.10991 |
| GIF | -0.63541 | 7.075051 | -3.64686 | 0.000298 | 0.002547 | -0.17198 |
| H19 | 0.66998 | 9.925031 | 3.604917 | 0.000349 | 0.002895 | -0.31728 |
| CXCL13 | -0.50063 | 8.267871 | -3.54847 | 0.00043 | 0.003414 | -0.51031 |
| KLK11 | 0.574516 | 8.204129 | 3.52677 | 0.000466 | 0.003651 | -0.58376 |
| C2orf40 | 0.659966 | 7.933045 | 3.449414 | 0.000617 | 0.004584 | -0.84212 |
| KIAA0367 | 0.555693 | 8.761755 | 3.441038 | 0.000636 | 0.004702 | -0.86977 |
| DES | 0.688745 | 8.405604 | 3.403118 | 0.000728 | 0.005227 | -0.99414 |
| DMN | 0.510893 | 8.341729 | 3.327062 | 0.000953 | 0.006492 | -1.23967 |
| C6orf58 | -0.68503 | 7.328375 | -3.22964 | 0.001334 | 0.008453 | -1.54649 |
| GP2 | -0.52445 | 8.076902 | -3.14512 | 0.001775 | 0.010603 | -1.8056 |
| SERPINA3 | 0.573419 | 11.3022 | 3.121613 | 0.001919 | 0.011244 | -1.87651 |
| PGA4 | -0.53366 | 6.507843 | -2.95943 | 0.003251 | 0.017173 | -2.35184 |
| GKN2 | -0.81488 | 8.207686 | -2.90396 | 0.003873 | 0.019637 | -2.50883 |
| REG3A | -0.74245 | 9.380015 | -2.81942 | 0.005032 | 0.024053 | -2.74259 |
| GKN1 | -0.80676 | 8.411593 | -2.72885 | 0.006614 | 0.029733 | -2.98565 |
| LOC644844 | -0.64047 | 10.43714 | -2.70193 | 0.007165 | 0.031623 | -3.05641 |
| PGA3 | -0.61501 | 7.273095 | -2.62987 | 0.008846 | 0.037118 | -3.2425 |
| PGA5 | -0.65168 | 7.428492 | -2.52795 | 0.011828 | 0.046223 | -3.4974 |
